# Significance of Epidermal Growth Factor Receptor (EGFR) upregulation in the prediction of the malignant transformation risk in oral potentially malignant disorders: a systematic review and meta-analysis

**DOI:** 10.3389/froh.2025.1578561

**Published:** 2025-03-27

**Authors:** José Luis Cívico-Ortega, Pablo Ramos-García, Miguel Ángel González-Moles

**Affiliations:** School of Dentistry, University of Granada. Biohealth Research Institute, Ibs.Granada, Granada, Spain

**Keywords:** EGFR, epidermal growth factor receptor, oral potentially malignant disorders, oral leukoplakia, malignant transformation, oral cancer, systematic review, meta-analysis

## Abstract

**Objectives:**

The purpose of this systematic review and meta-analysis was to appraise, both quantitatively and qualitatively, the extant evidence regarding the role of EGFR upregulation in predicting malignant transformation risk associated with oral potentially malignant disorders (OPMD).

**Methods:**

A comprehensive search was undertaken in the Web of Science, Embase, MEDLINE (via PubMed), and Scopus databases for longitudinal primary-level articles, whether prospective or retrospective in design, without restrictions on language or publication date. The QUIPS tool was employed for the purpose of assessing the potential for bias. A meta-analysis was conducted in addition to sensitivity analyses and analyses of the potential influence of small-study effects.

**Results:**

In total, eight studies, which were treated as nine distinct units for analytical purposes, were included in the final sample, which encompassed 653 patients with OPMD with follow-up data. EGFR upregulation was found to be significantly associated with an elevated malignant transformation risk of OPMD (RR = 2.17, 95%CI = 1.73–2.73, *p* < 0.001). Subgroup analyses demonstrated that both EGFR protein overexpression (RR = 2.02, 95%CI = 1.55–2.63, *p* < 0.001) and EGFR gene amplification (RR = 2.70, 95%CI = 1.72–4.25, *p* < 0.001), nuclear staining (RR = 3.47, 95%CI = 1.50-8.01, *p* = 0.004) and the >10% cutoff point were significantly associated with transformation risk (RR = 2.27, 95%CI = 1.33–3.87, *p* = 0.003).

**Conclusion:**

The present systematic review and meta-analysis demonstrates that EGFR overexpression, assessed through immunohistochemical technique, functions as a risk marker of OPMD malignant transformation risk.

**Systematic Review Registration:**

https://www.crd.york.ac.uk/PROSPERO/view/CRD42024626482, identifier: CRD42024626482).

## 1 Introduction

Oral potentially malignant disorders (OPMDs) are a group of lesions affecting the oral mucosa that involve an increased risk of developing oral cancer in patients affected by them, extending over the patient's lifetime, to all areas of the patient's oral mucosa—whether or not they are affected by the OPMD—and to other areas of their body, with priority being given to the upper aerodigestive tree (1). Oral squamous cell carcinoma (OSCC), the main malignancy resulting from malignant transformation of OPMDs, is a common and aggressive neoplasm. According to recent GLOBOCAN statistics (IARC, WHO) (2), the worldwide incidence of oral cancer is estimated to be 377,713 new cases per year, with approximately 177,757 deaths, resulting in a 5-year mortality rate of nearly 50% of cases. A relevant fact in this regard is that this mortality rate has not decreased substantially in the last 50 years (3). Today we know that reducing this mortality rate is very complex, probably due to the diversity of factors that affect it -factors derived from the patient, the tumor itself, the health institutions and the health policies of the different countries, as well as the economic and social development of the states- (4) and there is general agreement that the best way to reduce mortality from oral cancer is related to early diagnosis of the disease (5). In this sense, the care of patients with OPMDs and their follow-up would help in making an early diagnosis. However, most OPMDs do not progress to cancer (1), and it is a challenge for the clinician to reliably predict which OPMDs have a significantly increased risk of developing into carcinoma.

In recent years, there has been a remarkable amount of research on molecular alterations reported in OPMDs (6, 7), which could be considered as early events of oral carcinogenesis and thus, consequently, as markers of risk of malignancy in these OPMDs. Some of these molecules are currently considered as hallmarks of cancer published by Hanahan and Weinberg (8, 9), that is, molecules whose functions are considered to be distinctive of a cancer cell. One of these functions acquired by tumor cells is the ability to maintain proliferative signaling is of notable relevance during oral oncogenesis (10), and in this regard, the epidermal growth factor receptor (EGFR) has received much singular attention (11). EGFR (ErbB1/HER1) is the type receptor of the EGFR receptor family, which also includes ErbB2/HER2/Neu, ErbB3/HER3 and ErbB4/HER4 (12). The best known ligand of EGFR is EGF, although the receptor can also be activated by ligands such as TGF-α or HB-EGF, among others (13). The receptor can be constitutively activated by gene amplification or mutations, leading to complex EGFR-mediated signal transduction with regulation of downstream molecular signaling pathways, the most relevant being MAPK (ras-Raf-MEK-Erk) and PI3 K (PI3k-Akt-mTor) (14). These pathways culminate in actions that stimulate cell proliferation through the activation of transcription factors with positive regulation of important oncogenes (CCND1/cyclin D1), among the most relevant ones (15). EGFR overexpression has been listed as a poor prognostic factor in head and neck, esophageal, ovarian, cervical and bladder cancers through primary level studies (16–21). Moreover, the relevant oncogenic implications of EGFR have justified its consideration as a molecular target, with cetuximab being the first monoclonal antibody approved by the FDA for the treatment of head and neck cancer (22).

Despite the aforementioned facts, we must recognize that to date there are no studies with evidence-based designs, in the form of systematic reviews and meta-analyses, that analyze the role of EGFR alterations in OPMDs, and consequently it is not known whether the activation of the EGFR gene or the overexpression of its EGFR product, by mediating the acquisition of a distinctive feature of cancer -the ability to maintain a sustained proliferative signal- could behave as risk markers for the development of OSCC in OPMDs. The present study presents the results of a systematic review and meta-analysis of primary level studies selected on the basis of high methodological quality parameters that, in all cases, have followed patients over time, thus establishing a real association between malignant transformation of an OPMD and EGFR upregulation/overexpression.

## 2 Materials and methods

This systematic review with meta-analysis was conducted in accordance with the standards set forth by the MOOSE (23) and PRISMA (24) guidelines for structured reporting, as well as the principles established by the *Cochrane Collaboration* methodological criteria (25) and by *Cochrane Prognosis Methods Group* (26). The methodology for this study was developed in advance and recorded in PROSPERO, an international database of prospectively registered systematic reviews (registration number: ID- CRD42024626482). This approach was adopted in order to ensure transparency, precision, and integrity, with the subsequent mitigation of potential biases. The original comprehensive version of the protocol was developed in alignment with PRISMA-P standards to guarantee methodological rigour (27).

### 2.1 Search strategy

To identify pertinent sources, a systematic search was conducted across four databases: Web of Science, Embase, MEDLINE (via PubMed), and Scopus were consulted. The objective was to identify any relevant studies published prior to the upper date limit of November-2024, with no limitations regarding the lower date of publication. A mixture of thesaurus terms, including Medical Subject Headings (MeSH) and Emtree, combined with free-text terms (Supplementary Appendix S1) was used to enhance the sensitivity of the search strategy. Additionally, the list of references from the initially identified studies was subjected to a detailed examination. The management of references and the removal of duplicates were conducted employing Mendeley, version 1.19.8 (Elsevier, Amsterdam, The Netherlands).

### 2.2 Eligibility criteria

For inclusion in this study, publications were required to fulfill the criteria outlined below: (1) The publications could be in any language or publication year; (2) The study population consisted of patients suffering from any oral potentially malignant disorder (OMPD) (1); (3) EGFR upregulation was analyzed through protein overexpression -via immunohistochemistry- and/or gene amplification -via comparative genome hybridization, fluorescent *in situ* hybridization, Quantitative microsatellite analysis, BAC end sequencing, or digital karyotyping- (28); (4) The primary-level studies had to include data on the potential for malignant transformation, including cases involving both progression and non-progression towards oral cancer; 5) The studies needed to be designed as prospective or retrospective cohorts of longitudinal nature (29).

The exclusion criteria were as follows: (1) Articles that have been retracted, meta-analyses, reviews, editorials, case reports, letters, meeting abstracts, comments, book chapters and personal opinions; (2) Studies that have been conducted *in vitro* or *in animal* models; (3) Research on EGFR gene modifications other than amplification; (4) Studies on OSCC missing essential data regarding malignant transformation or related OPMDs; (5) Interventional or cross-sectional study designs; (6) Research studies lacking adequate statistical data to compute relative risk (RR) with 95% confidence intervals (CI); and (7) Articles that include the same population, as identified through author and affiliation verification, patient source confirmation, and examination of recruitment periods.

### 2.3 Study selection process

In applying the eligibility criteria, the two researchers (JLCO and PRG) worked independently and addressed any discrepancies through consensus. The selection process was conducted carried out across two distinct stages. To identify studies meeting the inclusion criteria, all titles and abstracts were initially subjected to a screening process. Subsequently, the full texts were reviewed to verify this fulfillment. Prior to undertaking these tasks, the evaluators underwent training and calibration in regard to ensuring consistency and reliability. In the screening phase, the reviewers initially assessed the articles jointly and got calibrated, employing the Cohen's kappa statistic to quantify inter-rater agreement. This resulted in near-perfect agreement (99.97%, kappa value = 0.99).

### 2.4 Data extraction

Data extraction from the sampled articles was independently performed by all authors (JLCO, PRG and MAGM), who completed a standardized data collection form using Excel (version 16/2018, Microsoft, Redmond, WA). Any differences were resolved through a consensus process. In accordance with the methodologies delineated by Luo et al. (2018) and Wan et al. (2014), the data, initially presented in order statistics, including the median, interquartile range, and minimum and maximum values, were subsequently transformed into means ± standard deviation (SD) (30, 31). In instances where merging datasets expressed as means ± SD from multiple subgroups into a single group was necessary, the formula recommended in the Cochrane Handbook was used (25). In studies that examined multiple alterations, such as amplification and overexpression, each dataset was collected and analyzed independently. The collected data included the following details: first author's name, year of publication, language of the study, study design, geographical region (country and continent), and recruitment and follow-up periods. Additional variables included the sample size, the anatomical site of the lesions, patient demographics (age and sex), as well as information on tobacco and alcohol use. Furthermore, the dataset covered the type of OPMD, characteristics of oral epithelial dysplasia, progression to oral cancer, and the upregulation assessed (EGFR overexpression or *EGFR* amplification). For the studies that utilized immunohistochemistry, the retrieved data included the established cutoff points, scoring systems, and the reported proportion of cases exhibiting EGFR overexpression.

### 2.5 Evaluation and risk of bias

A pair of researchers (JLCO and PRG), in accordance with the recommendations of the Cochrane Prognosis Methods Group, employed the QUIPS tool (Quality in Prognosis Studies) to conduct a critical appraisal of the quality and potential for bias in the included studies (32). This entailed an analysis of six domains of potential bias, as follows: (1) Study participation, (2) Study attrition, (3) Measurement of prognostic factors, (4) Outcome measurement, (5) Study confounding, and (6) Statistical analysis and reporting. Each domain was evaluated according to a scoring system comprising three levels of concern: low, moderate, and high. The overall score was determined based on the two critical domains (No. 3 and No. 5) using a previously established method by our researching group (27, 33–36). The objective was to conduct a statistical analysis to ascertain the influence of methodological rigour on the findings of our primary-level studies meta-analysis.

### 2.6 Statistical analysis

The analysis of EGFR upregulation (i.e., protein overexpression or gene amplification) was carried out in accordance with the scoring systems adopted by primary-level studies, with results expressed as a dichotomous categorical variable. The primary-level studies were used to compute relative risks (RRs) along with their 95% confidence intervals (CIs). Afterwards, these were combined using the inverse variance method under a random-effects model. This approach, which is based on the classical DerSimonian and Laird method, was designed to address potential variability across different study subpopulations, including variations in OPMDs, EGFR detection techniques, and affected oral subsites.

The extent of heterogeneity among the selected articles was evaluated employing Cochran's *Q*-test. Given the limited statistic capacity of this assessment tool, a significance level of *p* < 0.10 was employed to ensure a reasonable degree of statistical rigor. To provide a further quantification of inter-study heterogeneity, Higgins' *I*^2^ statistic was employed. This statistic represents the proportion of observed variance in effect sizes that can be attributed to genuine differences in effect, as opposed to sampling error. A value between 50% and 75% was deemed to suggest a moderate-to-high level of heterogeneity across studies (37, 38).

The objective of the study was to ascertain any possible heterogeneity sources and to investigate the key factors linked to EGFR overexpression and malignant transformation in OPMDs. To achieve this goal, planned subgroup meta-analyses were conducted.

A forest plot was constructed for each meta-analysis with the intention of providing a graphical representation of the overall effects, thus facilitating successive visual inspection. Subsequently, to evaluate the stability and reliability of the meta-analysis outcomes, secondary analyses were conducted, including sensitivity analyses, to assess the impact of individual studies on the aggregated estimate (39). This was accomplished by employing a “leave-one-out” approach, whereby the meta-analysis was repeated in sequence with each study omitted individually. Funnel plots were constructed to evaluate the influence of small-study effects, including publication bias, in the final analysis. To this end, the Egger regression test was applied. (pEgger < 0.10 was regarded as a noteworthy asymmetry) (40). All statistical data were analyzed using the Stata software (version 16.1, Stata Corp, College Station, TX, USA).

## 3 Results

### 3.1 Results of the literature search

**Figure 1:**
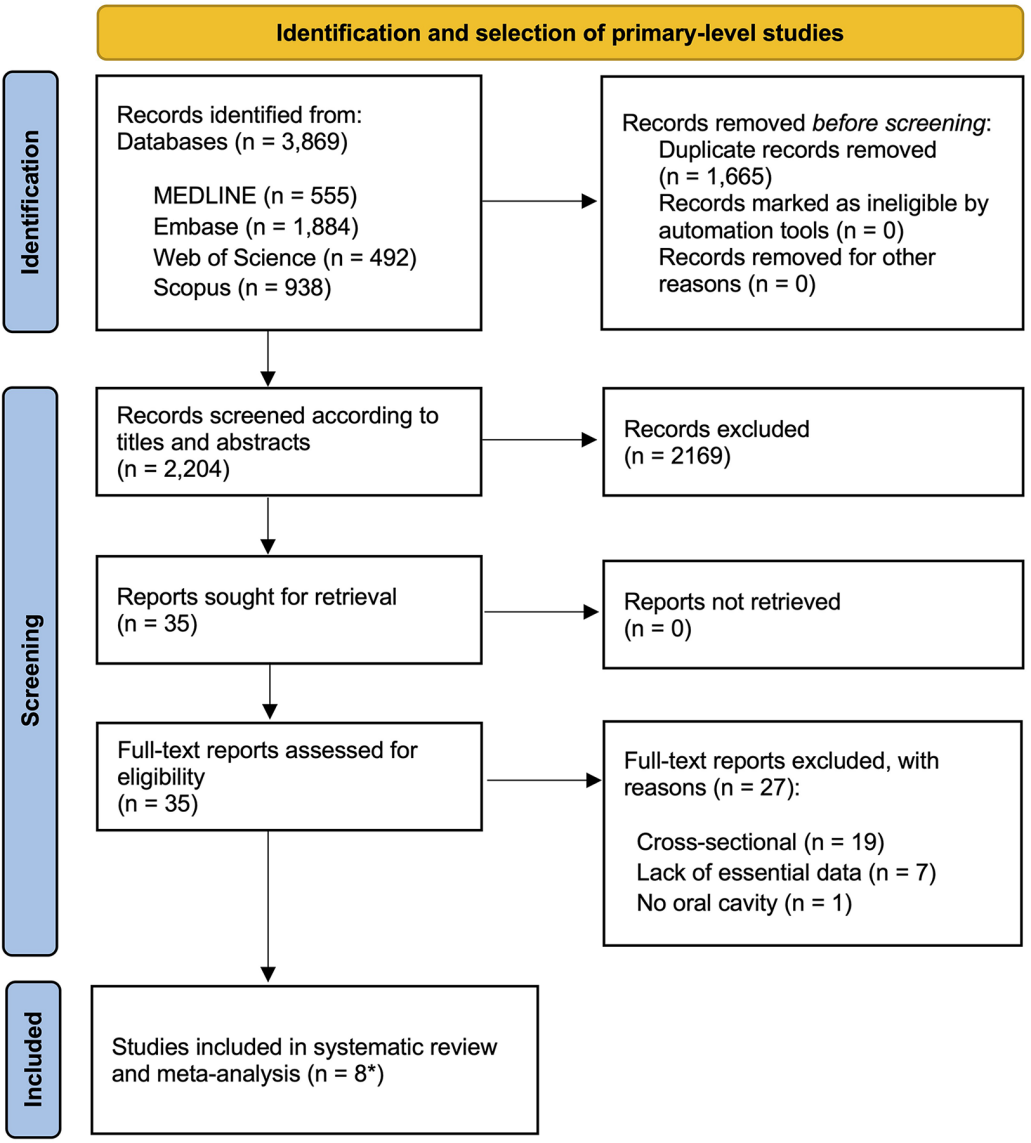
Flow diagram showing the identification and selection process of relevant studies, analyzing the implications of EGFR upregulation in the prediction of the malignant transformation risk of oral potentially malignant disorders (OPMD). *eight primary-level studies systematically reviewed and meta-analyzed as nine different units of analysis.

The flow diagram in Figure 1 provides a visual representation of the process undertaken to identify and select studies for inclusion in the review. The final count totalled 3,869 records, comprising 1,884 from Embase, 938 from Scopus, 555 from PubMed, 492 from the Web of Science, with one additional record sourced from a manual review of reference lists from the initial set of studies. Following the elimination of duplicates, the remaining 2,204 studies were subjected to screening based on their titles and abstracts. Of the 35 papers initially deemed eligible for consideration, it was determined that 27 did not fully align with the established criteria. Supplementary Appendix S6 contains a list of the excluded studies, along with the reasons for their exclusion. Ultimately, the final sample consisted of eight studies -analysed as nine different analysis units because one study identified EGFR upregulation by protein overexpression and gene amplification- that were selected for both quantitative and qualitative appraisal (41–48).

### 3.2 Study characteristics

Table 1 provides an overview of the key characteristics of the selected papers, whereas a detailed account of the attributes of each primary-level study is provided in Supplementary Appendix S2. A total of 653 patients with OPMD were recruited for the nine studies published between 2007 and 2022, which provided data on malignant transformation. The sample size for each study varied between 23 and 145 participants. Six studies evaluated EGFR overexpression, while three assessed EGFR amplification. The selected articles were conducted in three main regions: Asia (*n* = 1), North America (*n* = 3), and Europe (*n* = 5). Of the nine studies included, seven were retrospective in design, while the remaining two were prospective. Immunohistochemistry was the predominant method used, employing a variety of anti-EGFR antibodies, including clones 111.6, 31G7, EGFR-1, EGFR.25, and M3563, each reported in a single study. Further details regarding the immunohistochemical methods are provided in Table 1.

**Table 1:** Summarized study characteristics*.*

Summarized characteristics of the study sample	
Total	9 studies^a^
Year of publication	2,007–2022
Total patients (range)	653 (23—148)
Type of OPMDs	
Oral leukoplakia	3 studies
Mixed	3 studies
Not reported	3 studies
Study design	
Retrospective	7 studies
Prospective	2 studies
Experimental methods for EGFR upregulation determination	
Protein overexpression (Immunohistochemistry)	6 studies
Gene amplification (FISH)	3 studies
Anti-EGFR antibody	
Clone 111.6	1 study
Clone 31G7	1 study
Clone EGFR-1	1 study
Clone EGFR.25	1 study
M3563	1 study
Not applicable (gene amplification)	3 studies
Not reported	1 study
Anti-EGFR antibody dilution	
<1:100	2 studies
≥1:100	3 studies
Not applicable (gene amplification)	3 studies
Not reported	1 study
Anti-EGFR antibody incubation time	
Overnight	1 study
90´	2 studies
Not applicable (gene amplification)	3 studies
Not reported	3 studies
Anti-EGFR antibody incubation temperature	
4°C	1 study
Room temperature	1 study
Not applicable (gene amplification)	3 studies
Not reported	4 studies
Cut-off point	
10%	2 studies
>10%	2 studies
Intensity	1 study
Not applicable (gene amplification)	3 studies
Not reported	1 study
Immunostaining pattern	
Membrane/cytoplasm	5 studies
Nuclear	1 study
Not applicable (gene amplification)	3 studies
Geographical region	
Asia	1 study
Europe	5 studies
North America	3 studies

^a^ note: Eight primary-level studies, published and meta-analyzed as nine different units of analysis.

### 3.3 Qualitative evaluation

**Figure 2:**
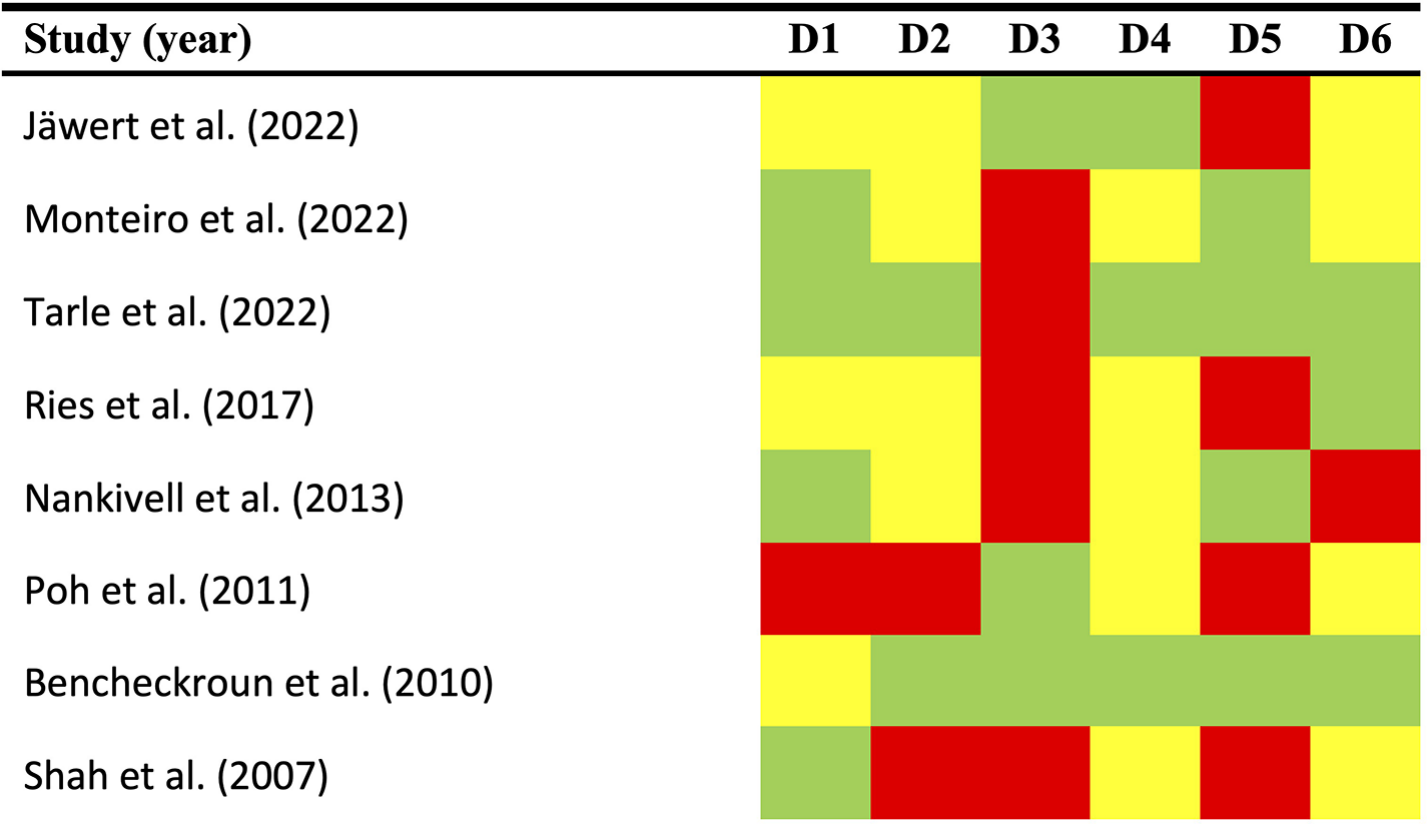
Quality plot graphically representing the risk of bias (RoB) across primary-level studies using a method specifically designed for systematic reviews and meta-analyses addressing questions on prognostic factor studies (i.e., Quality in Prognosis Studies -QUIPS- tool, developed by members of the Cochrane Prognosis Methods Group). The following domains (D1–D6) were critically judged: D1, study participation; D2, study attrition; D3, prognostic factor measurement; D4, outcome measurement; D5, study confounding; and D6, statistical analysis/reporting. RoB was assessed for all domains throughout all studies and scored as potentially low (depicted as green color), moderate (yellow color), or high (red color).

To ensure the highest standards of quality, we conducted a comprehensive qualitative analysis, evaluating potential sources of bias across six domains using the Quality In Prognosis Studies (QUIPS) tool (Figure 2):

*Study participation (D1).* A total of 50% of the studies were deemed to have a low risk of bias, 37.5% had a moderate risk, and 12.5% had a high risk (Figure 2). The most prevalent biases were an absence of sufficient reporting on patient demographic and clinical information (such as age, sex, and affected subsites), as well as a lack of reporting on the study period and place of recruitment.

*Study attrition (D2).* A low risk was identified in 25% of instances, a moderate risk was evident in 25%, and a high risk of bias was identified in 50% (Figure 2). Insufficient detail in the reporting of essential information, such as the follow-up period duration (expressed as mean, SD, or estimable order statistics like median, IQR, or range) or details on dropout rates, represented the most frequent sources of bias.

*Prognostic factor measurement (D3)*. Potential bias was identified as low in 37.5% of the studies evaluated, and high in 62.5% (Figure 2). It is noteworthy that the most prevalent form of risk of bias is also among the most crucial, namely the optimised cut-off point. Furthermore, enhancements to the reporting information concerning the immunohistochemical technique and scoring methods are also recommended.

*Outcome measurement (D4)*. The risk of bias was identified as low in 37.5% of cases, in the remaining 62.5% of instances, it was determined to be moderate. (Figure 2). The highest score was awarded for this domain due to the universal acceptance of the clinical and histopathological methods employed for OSCC diagnosis. Details about the system used were not provided, which did not result in a quality or risk of bias assessment. However, potential bias may still be present in this domain, due to the limited follow-up period. The length of the follow-up period is a critical element in longitudinal studies, as it allows for the observation of noteworthy events to take place.

*Study confounding (D5)*. A 50% low-risk bias prevalence was observed across the studies reviewed, with the remaining 50% demonstrating high-risk bias. The most common source of bias was the inadequate consideration or incorrect measurement of confounding factors. The studies did not define any factors *a priori* that were regarded as potential confounders. Furthermore, the studies did not subsequently discuss the probable biological interactions involving EGFR upregulation and the aforementioned factors.

*Statistical analysis and reporting (D6)*. A high risk of bias was observed in 12.5% of the studies included in the systematic review, while 50% demonstrated a moderate risk and 37.5% exhibited a low risk. The most frequently occurring form of bias was a lack of reporting of relative risk data accompanied by a 95% confidence interval, as well as a tendency towards selective reporting of data.

*Overall quality.* Most studies were found to be acceptable, with a range of results across the various domains. According to the established scoring system, which takes into account potential limitations of critical domains, an overall high risk of bias was identified in two articles (42, 46), while six primary-level studies were critically judged as an overall low risk of bias (41, 43–45, 47, 48).

Recommendations for future studies include recording comprehensive characteristics from participants to ascertain the representativeness of the study sample, as well as for follow-up period and patient drop-out rate (D1 and D2). It is essential to use standardised cut-off points, rather than optimized cut-off points, be employed in addition to the reporting of the details of the IHC technique and scoring methods (D3). Furthermore, it is necessary to design and implement follow-up periods that are sufficiently long to allow the study event (i.e., malignant transformation) to elapse (D4). It is also advised the conduction of multivariable analyses in order to control for confounding variables (D5). Finally, it is essential to avoid selective reporting of data and to utilize appropriate effect size measures with their corresponding confidence intervals (D6).

### 3.4 Quantitative evaluation

#### 3.4.1 Meta-analysis on the association between EGFR upregulation and OPMDs malignant transformation risk

**Figure 3:**
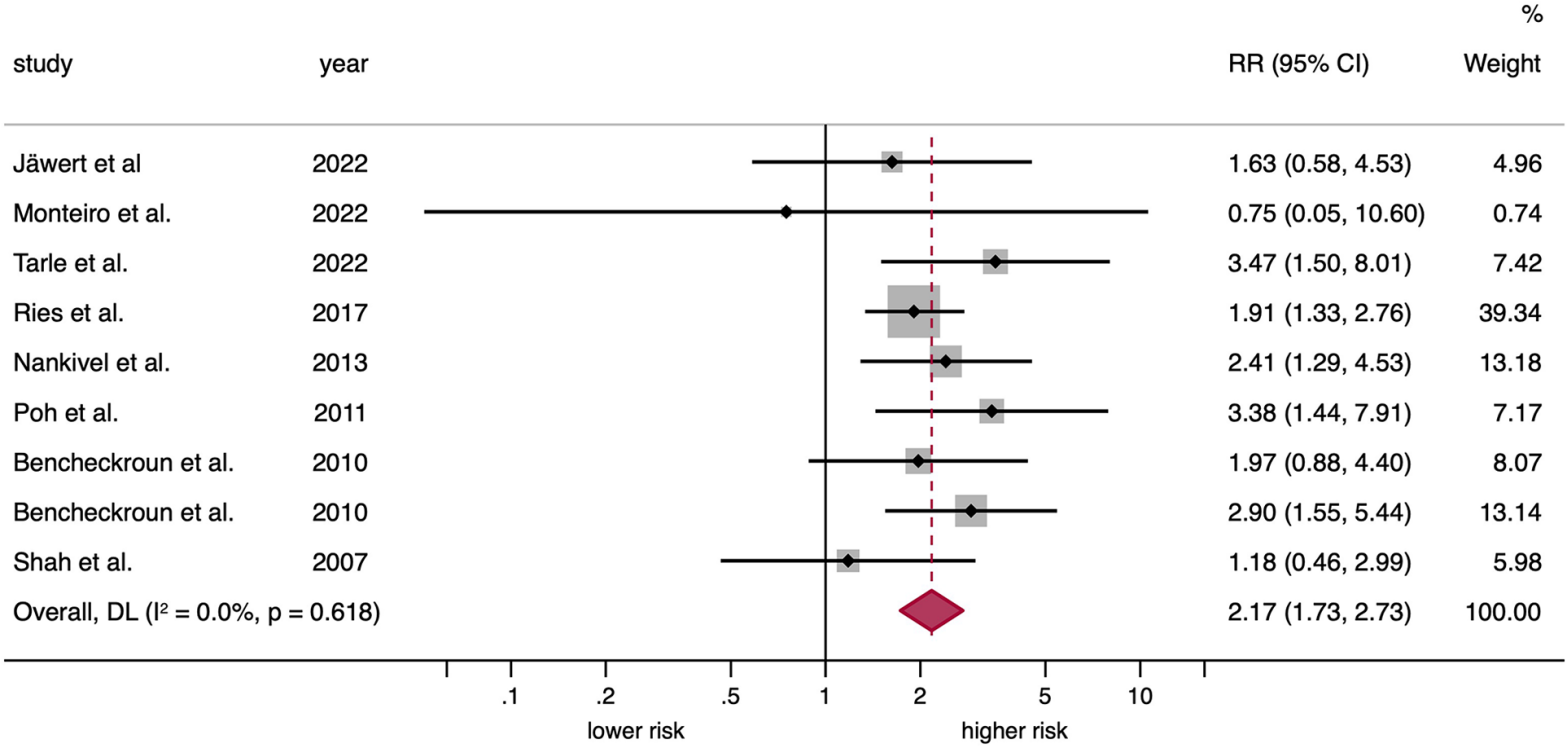
Forest plot graphically representing the meta-analysis on the association between EGFR upregulation and malignant transformation risk in patients with OPMD. Random-effects model, DL inverse-variance weighting. Diamonds indicate the pooled RRs with their corresponding 95%CIs. EGFR, epidermal growth factor receptor; OPMD, oral potentially malignant disorders; RR, relative risk; CI, confidence intervals; DL, DerSimonian and Laird. eight primary-level studies entered into meta-analysis as nine different units of analysis, due to one study (Benchekroun et al. 2010) reported EGFR upregulation through protein overexpression and gene amplification.

A significantly elevated risk was identified among patients with OPMDs and EGFR upregulation when a random-effects model was employed (RR = 2.17, 95% CI = 1.73–2.73, *p* < 0.001). Moreover, no substantial level of interstudy heterogeneity was observed (*p* = 0.62, *I*^2^ = 0.0%; Figure 3, Table 2).

**Table 2:** meta-analyses of the predictive value of EGFR upregulation on the malignant transformation risk of OPMD.

Meta-analyses	No. of studies	No. of patients	Stat. Model	Wt	Pooled data		Heterogeneity	
					ES (95% CI)	*P*-value	*P*_het_	*I^2^* (%)
Malignant transformation risk^a^	9^d^	653	REM	D-L	RR = 2.17 (1.73–2.73)	<0.001	0.62	0.0
Subgroup analysis by alteration^b^						0.28^c^		
EGFR protein overexpression	6	546	REM	D-L	RR = 2.02 (1.55–2.63)	<0.001	0.58	0.0
*EGFR* gene amplification	3	106	REM	D-L	RR = 2.70 (1.72–4.25)	<0.001	0.53	0.0
Subgroup analysis by geographical region^b^						0.26^c^		
Asia	1	60	REM	D-L	RR = 1.18 (0.46–3.00)	0.73	—	0.0
Europe	5	364	REM	D-L	RR = 2.09 (1.58–2.77)	<0.001	0.62	0.0
North America	3	229	REM	D-L	RR = 2.70 (1.76–4.14)	<0.001	0.64	0.0
Subgroup analysis by type of OPMD^b^						0.39^c^		
Leukoplakia	3	166	REM	D-L	RR = 1.85 (1.31–2.59)	<0.001	0.77	0.0
Mixed	3	258	REM	D-L	RR = 2.24 (1.29–3.86)	0.004	0.23	31.4
Not reported	3	229	REM	D-L	RR = 2.70 (1.76–4.14)	<0.001	0.64	0.0
Subgroup analysis by immunohistochemical pattern^b^						0.23^c^		
Membrane/cytoplasm	5	496	REM	D-L	RR = 1.90 (1.44–2.51)	<0.001	0.73	0.0
Nuclear	1	50	REM	D-L	RR = 3.47 (1.50–8.01)	0.004	—	0.0
Not applicable (gene amplification)	3	107	REM	D-L	RR = 2.70 (1.72–4.25)	<0.001	0.53	0.0
Subgroup analysis by anti-EGFR antibody^b^						0.54^c^		
Clone 111.6	1	60	REM	D-L	RR = 1.18 (0.46–3.00)	0.73	—	0.0
Clone 31G7	1	145	REM	D-L	RR = 1.97 (0.88–4.40)	0.10	—	0.0
Clone EGFR-1	1	50	REM	D-L	RR = 3.47 (1.50–8.01)	0.004	—	0.0
Clone EGFR.25	1	45	REM	D-L	RR = 0.75 (0.05–10.61)	0.83	—	0.0
M3563	1	98	REM	D-L	RR = 1.91 (1.33–2.74)	<0.001	—	0.0
Not applicable (gene amplification)	3	107	REM	D-L	RR = 2.70 (1.72–4.25)	<0.001	0.53	0.0
Not reported	1	148	REM	D-L	RR = 2.41 (1.29–4.52)	0.006	—	0.0
Subgroup analysis by anti-p53 antibody dilution^b^						0.64^c^		
<1:100	2	110	REM	D-L	RR = 2.06 (0.72–5.93)	0.18	0.03	64.9
≥ 1:100	3	288	REM	D-L	RR = 1.89 (1.36–2.63)	<0.001	0.79	0.0
Not applicable (gene amplification)	3	107	REM	D-L	RR = 2.70 (1.72–4.25)	<0.001	0.53	0.0
Not reported	1	148	REM	D-L	RR = 2.41 (1.29–4.52)	0.006	—	0.0
Subgroup analysis by anti-EGFR antibody incubation time^b^						0.37^c^		
Overnight	1	60	REM	D-L	RR = 1.18 (0.46–3.00)	0.73	—	0.0
90´	2	195	REM	D-L	RR = 2.58 (1.45–4.61)	0.001	0.34	0.0
Not applicable (gene amplification)	3	107	REM	D-L	RR = 2.70 (1.72–4.25)	<0.001	0.53	0.0
Not reported	3	291	REM	D-L	RR = 2.00 (1.46–2.73)	<0.001	0.63	0.0
Subgroup analysis by anti-EGFR antibody incubation temperature^b^						0.46^c^		
4°C	1	60	REM	D-L	RR = 1.18 (0.46–3.00)	0.73	—	0.0
Room temperature	1	145	REM	D-L	RR = 1.97 (0.88–4.40)	0.10	—	0.0
Not applicable (gene amplification)	3	107	REM	D-L	RR = 2.70 (1.72–4.25)	<0.001	0.53	0.0
Not reported	4	341	REM	D-L	RR = 2.13 (1.59–2.86)	<0.001	0.49	0.0
Subgroup analysis by cutoff point for EGFR protein overexpression^b^						0.62^c^		
10%	2	205	REM	D-L	RR = 1.58 (0.86–2.91)	0.14	0.41	0.0
>10%	2	148	REM	D-L	RR = 2.27 (1.33–3.87)	0.003	0.20	39.2
Intensity	1	45	REM	D-L	RR = 0.75 (0.05–10.61)	0.83	—	0.0
Not applicable (gene amplification)	3	107	REM	D-L	RR = 2.70 (1.72–4.25)	<0.001	0.53	0.0
Not reported	1	148	REM	D-L	RR = 2.41 (1.29–4.52)	0.006	—	0.0
Subgroup analysis by overall risk of bias in primary-level studies^b^						0.13^c^		
Low RoB	7	495	REM	D-L	RR = 2.55 (1.87–3.47)	<0.001	0.80	0.0
High RoB	2	158	REM	D-L	RR = 1.79 (1.28–2.51)	0.001	0.35	0.0

Stat. Model, statistical model; Wt, method of weighting; ES, effect size estimation; RR, relative risk; CI, confidence intervals; REM, random-effects model; D-L, DerSimonian and Laird method; OPMD, oral potentially malignant disorders; RoB, risk of bias; ED, oral epithelial dysplasia.

^a^ Meta-analysis of aggregate (summary) data.

^b^ Subgroup meta-analyses.

^c^ Test for between-subgroup differences.

^d^ Eight primary-level studies, published and meta-analyzed as nine different units of analysis.

#### 3.4.2 Subgroups meta-analysis

The observed association remained significant across several subgroups—potentially representing sources of heterogeneity or exhibiting a substantial effect size- and were defined according to type of alteration (EGFR protein overexpression: RR = 2.02, 95% CI = 1.55–2.63, *p* < 0.001; *EGFR* gene amplification: RR = 2.70, 95% CI = 1.72–4.25, *p* < 0.001); immunohistochemical pattern (nuclear: RR = 3.47, 95% CI = 1.50–8.01, *p* = 0.004; membrane/cytoplasmic: RR = 1.90, 95% CI = 1.44–2.51, *p* < 0.001); by the specific anti-EGFR antibody utilized (Clone EGFR-1: RR = 3.47, 95% CI = 1.50–8.01, *p* = 0.004; M3563: RR = 1.91, 95% CI = 1.33–2.74, *p* < 0.001; Clone 31G7: RR = 1.97, 95% CI = 0.88–4.40, *p* = 0.10; Clone EGFR.25: RR = 0.75, 95% CI = 0.05–10.61, *p* = 0.83; Clone 111.6: RR = 1.18, 95% CI = 0.46–3.00, *p* = 0.73); geographical location (North America: RR = 2.70, 95% CI = 1.76–4.14, *p* < 0.001; Europe: RR = 2.09, 95% CI = 1.58–2.77, *p* < 0.001; Asia: RR = 1.18, 95% CI = 0.46–3.00, *p* = 0.73); type of OPMD (mixed: RR = 2.24, 95% CI = 1.29–3.86, *p* = 0.004; leukoplakia: RR = 1.85, 95% CI = 1.31–2.59, *p* < 0.001); by incubation temperature (room temperature: RR = 1.97, 95% CI = 0.88–4.40, *p* = 0.10; 4°C: RR = 1.18, 95% CI = 0.46–3.00, *p* = 0.73); by cutoff point (>10%: RR = 2.27, 95% CI = 1.33–3.87, *p* = 0.003; 10%: RR = 1.58, 95% CI = 0.86–2.91, *p* = 0.14; intensity: RR = 0.75, 95% CI = 0.05–10.61, *p* = 0.83); by incubation time (90': RR = 2.58, 95% CI = 1.45–4.61, *p* = 0.001; overnight: RR = 1.18, 95% CI = 0.46–3.00, *p* = 0.73); and by dilution (≥1:100: RR = 1.89, 95% CI = 1.36–2.63, *p* < 0.001; <1:100: RR = 2.06, 95% CI = 0.72–5.93, *p* = 0.18) (Supplementary Appendix S3).

### 3.5 Quantitative secondary analyses

#### 3.5.1 Analysis of small-study effects

The funnel plot revealed no evidence of asymmetry (Supplementary Appendix S4). This finding was validated by the Egger test (p_Egger_ = 0.846), indicating that small-study effects, including potential publication bias, did not impact the results and strengthening their reliability.

#### 3.5.2 Sensitivity analysis

The sequential repetition of meta-analyses, conducted by systematically removing one study at a time through the “leave-one-out” method, yielded consistent overall results (Supplementary Appendix S5). This supports that the reported relative risk was not affected by the exclusion of any particular primary-level study, thereby confirming the stability of our findings.

## 4 Discussion

Our systematic review and meta-analysis on the risk of oral cancer development in patients with OPMDs as a function of EGFR upregulation, conducted on 9 primary level studies including 653 patients, demonstrates that OPMDs which carry upregulation of EGFR have a significantly increased risk of oral cancer development compared to those OPMDs in which EGFR is not upregulated (RR = 2.17, 95%CI = 1.73.2.73, *p* < 0.001). This increased risk of oral cancer is significantly higher both in OPMDs in which EGFR gene amplification was detected (RR = 2.70, 95%CI = 1.72–4.25, *p* < 0.001), assessed in 3 studies and 106 patients, in all cases using the FISH technique, and in OPMDs in which immunohistochemistry was used to assess EGFR protein overexpression (RR = 2.02, 95%CI = 1.55–2.63, *p* < 0.001). It should be noted that there were no significant differences in terms of cancer risk prediction dependent on the study of gene amplification vs. immunohistochemical detection of EGFR protein overexpression (*p* = 0.28), which is relevant since immunohistochemistry is an inexpensive technique routinely practiced in diagnostic pathology laboratories. EGFR upregulation indicates the establishment of a sustained hyperproliferative state in cells of the oral epithelium affected by OPMDs, which is a hallmark of tumor cells (8, 9) and points to EGFR as an early event in oral carcinogenesis and a marker of risk of oral cancer development in OPMDs, probably facilitating the establishment of genomic instability and with it the acquisition of new oncogenic summative events that conclude with the final development of invasive cell clones. A first deliverable of our study is that the detection of EGFR protein overexpression by immunohistochemical methods would be advisable in the assessment of the risk of cancer development in OPMDs.

The studies included in our meta-analysis focus essentially on oral leukoplakia (3 studies, 166 patients); three other primary level studies (258 patients) include cases of leukoplakia, erythroplakia and oral submucous fibrosis, while the remaining three studies (107 patients) do not specify the OPMDs analyzed. It should be pointed out that there is no information on some very relevant OPMDs due to their frequency -oral lichen planus- or due to their enormous tendency to develop cancer -proliferative verrucous leukoplakia- and consequently it would be advisable to carry out future studies that focus on the evaluation of the risk of developing oral cancer in oral lichen planus and proliferative verrucous leukoplakia based on EGFR upregulation. Oral leukoplakia, on which we have more information regarding the predictive value of EGFR, is probably the most relevant of all OPMDs due to its high prevalence in the general population -between 1.36% and 4.11%- (49–51) and to its high malignancy rate. A recent meta-analysis of our research group indicates that 6.64% of oral leukoplakia develop oral cancer (52). The risk of cancer development is especially elevated for non-homogeneous leukoplakia (RR = 4.23), large (RR = 2.08), located on the lateral border of the tongue vs. other intraoral locations (RR = 2.09), in patients with smoking (RR = 1. 64) and when they have developed epithelial dysplasia (RR = 2.75); we can also state based on the evidence that oral leukoplakias overexpressing EGFR present a risk of cancer development 1.85 times higher than those not overexpressing this protein (95%CI = 1.31–2.59, *p* < 0.001). Consequently, along with the other clinicopathologic parameters mentioned above that have demonstrated their value in predicting the risk of malignancy of oral leukoplakia, the analysis of immunohistochemical overexpression of EGFR is indicated for this purpose. It should also be noted that there were no geographical differences in relation to the predictive value of EGFR in OPMDs, with a very similar risk in studies performed in Europe (RR = 2.09) and North America (RR = 2.70) (*p* = 0.26), indicating that EGFR overexpression and the oncogenic mechanisms associated with this protein do not depend on other factors -e.g., etiological- associated with a particular geographical location in the world. There is limited knowledge regarding OPMDs in Asia, which demands further research in this area of the world.

Our work shows that EGFR increased the risk of progression to cancer of OPMDs regardless of the cellular location in which its overexpression was detected, either in the cell membrane and cytoplasm (RR = 1.90) or in the nucleus (RR = 3.47). This result indicates that both locations should be evaluated and, interestingly, that EGFR exerts oncogenic stimuli not only linked to its proliferation-stimulating actions via receptor effects, but also acting as a transcription factor, of which we know its capacity to stimulate important oncogenes with CCND1/cyclin D1, MYC, STAT3/5, Akt and ERK (53–55). Finally, we can also provide valuable information on the best cutoff point for EGFR overexpression for the consideration of a case as positive; we know that malignancies overexpressing EGFR in more than 10% of their epithelial cells have a 2.27-fold increased risk of developing oral cancer, which was significantly higher than the risk obtained with other lower cutoff points (*p* = 0.003).

Recently, some important systematic reviews from the World Workshop on Oral Medicine (WWOM) group (7) and from the WHO Collaborating Center for Oral cancer (6) have evaluated additional emerging molecular markers with translational potential in the prediction of malignant transformation risk of OPMD, specially focused on oral leukoplakias. Promising biomarkers were highlighted, such as podoplanin, a well-known regulator of the epithelial-mesenchymal-transition (EMT) phenomenon, which exerts oncogenic roles in human cancer, directly related with the acquisition of a migratory and proinvasive phenotype (56). Other relevant biomarkers were also pointed out, such as p53 and p27, with well-established major canonical functions implicated in the regulation of apoptosis, DNA damage repair and cell cycle control. Finally, important classical chromosomal loci abnormalities, singularly aneuploidy and loss of heterocigosity, were also found to be associated with the malignant transformation of oral leukoplakia. It has also been pointed out that none of the markers currently harbours enough evidence to be considered as definitive tools for predicting malignant transformation of oral leukoplakia (6, 7). It was also suggested the possibility to analyse the co-expression of multiple biomarkers combined taken together, in comparison to the use of a single biomarker (6). In this sense, future studies should evaluate EGFR overexpression in the context of other multiple biomarkers as a single variable, across multivariable models adjusted for potentially confounding factors.

The potential limitations of this systematic review and meta-analysis should be discussed. First, the absence of clinical and demographic data from primary-level studies should be highlighted. Key variables such as age, sex, tobacco and alcohol consumption, and presence and grade of epithelial dysplasia were not systematically reported, which limited the possibility of performing secondary analyses adjusted for these potential confounders. Nevertheless, these are truly inherent limitations from primary-level studies, so it is recommended that future primary-level studies focus on enhancing data reporting and methodological standardization. On the other hand, heterogeneity is a common concern in most systematic reviews and meta-analyses. However, it is not a limitation of the present meta-analytical study, in which we did not identify considerable sources of clinical, methodological or statistical heterogeneity, with no impact on the interpretation of the results, which are stable, reliable and robust.

A significant strength of this meta-analysis is the sample of primary-level studies comprised of longitudinal cohorts, which ensures the follow up of patients over time. This methodological approach guarantees higher quality of evidence compared to studies of a cross-sectional nature, as is typical of most investigations into other biomarkers in OPMD. Consequently, this study provides a more precise evaluation of causality and risk analysis, with the potential to offer conclusions that are more aligned with reality concerning the impact of EGFR upregulation and the malignant transformation risk of OPMDs.

In conclusion, our meta-analysis demonstrates that EGFR overexpression demonstrated by immunohistochemistry in the oral epithelium of OPMDs behaves as a risk marker for the development of oral cancer and consequently its evaluation using immunohistochemical methods would be advisable.

## Data Availability

The original contributions presented in the study are included in the article/Supplementary Material, further inquiries can be directed to the corresponding authors.

## Supplementary material

The Supplementary Material for this article can be found online https://www.frontiersin.org/articles/10.3389/froh.2025.1578561/full#supplementary-material
