# Supplementary material for: Significance of Epidermal Growth Factor Receptor (EGFR) upregulation in the prediction of the malignant transformation risk in oral potentially malignant disorders: a systematic review and meta-analysis
Source: Front Oral Health. 2025 Mar 27;6:1578561. doi: 10.3389/froh.2025.1578561 (PMC11983453; doi:10.3389/froh.2025.1578561)
Supplement: Supplementary file 1 [file Table1.docx]

**Appendix to the manuscript**

**Significance of Epidermal Growth Factor Receptor (EGFR) upregulation in the prediction of the malignant transformation risk in oral potentially malignant disorders: A systematic review and meta-analysis**

**Table of contents**

1. Supplementary Appendix S1. Search strategy 3

2. Supplementary Appendix S2. List of excluded studies with reasons 4

3. Supplementary Appendix S3. Descriptive characteristics of the study sample 8

4. Supplementary Appendix S4. Meta-analysis on the association between EGFR upregulation and OPMDs malignant transformation risk 9

5. Supplementary Appendix S5. Analysis of small‐study effects 19

6. Supplementary Appendix S6. Sensitivity analysis 20

**1. Supplementary Appendix S1. Search strategy**

**Search strategy for each database, number of results, and execution date.**

| **Database** | **Query/Search Strategy** | **Results/ Items found** | **Search time limits** |
| --- | --- | --- | --- |
| MEDLINE | ("Genes, erbB-1"[Mesh] OR "epidermal growth factor receptor"[all fields] OR "EGFR"[all fields] OR "erbb1"[all fields] OR "cerbb1"[all fields] OR "her1"[all fields] OR "pEGFR"[all fields] OR "phospho-EGFR"[all fields]) AND ("mouth"[MeSH Terms] OR "mouth"[All Fields] OR "oral"[All Fields] OR oropharyn*[All Fields]) AND ("carcinoma, squamous cell"[MeSH Terms] OR ("carcinoma"[All Fields] AND "squamous"[All Fields] AND "cell"[All Fields]) OR "squamous cell carcinoma"[All Fields] OR “dysplasia"[All Fields] OR “potentially malignant disorders”[All Fields] OR premalign*[All Fields] OR precancer*[All Fields] OR “leukoplakia”[All Fields] OR “erythroplakia”[All Fields] OR “lichen planus”[All Fields] OR “submucous fibrosis”[All Fields]) AND (“transformation”[All Fields] OR “progression”[All Fields] OR “risk”[All Fields]) | 555 | Nov,  2024 |
| Embase | ('epidermal growth factor receptor'/exp OR 'epidermal growth factor receptor' OR 'EGFR' OR 'erbb1' OR 'cerbb1' OR 'her1' OR 'pEGFR' OR 'phospho-EGFR') AND ('mouth' OR 'oral' OR 'oropharyn*') AND ('squamous cell carcinoma'/exp OR 'squamous cell carcinoma' OR ('squamous' AND ('cell'/exp OR 'cell') AND ('carcinoma'/exp OR 'carcinoma')) OR 'dysplasia' OR 'potentially malignant disorders' OR 'premalign*' OR 'precancer'/exp OR 'precancer*' OR 'leukoplakia'/exp OR 'leukoplakia' OR 'erythroplakia' OR 'erythroplakia' OR 'lichen planus'/exp OR 'lichen planus' OR 'submucous fibrosis') AND ('transformation' OR 'progression' OR 'risk') | 1,884 | Nov,  2024 |
| Web of Science | TS=("epidermal growth factor receptor" OR "EGFR" OR “erbb1” OR "cerbb1" OR "her1" OR "pEGFR" OR "phopho-EGFR") AND TS=(mouth OR oral OR oropharyn*) AND TS=(“squamous cell carcinoma”) AND TS=(transformation OR progression OR risk) | 492 | Nov,  2024 |
| Scopus | TITLE-ABS-KEY(("epidermal growth factor receptor" OR "EGFR" OR “erbb1” OR "cerbb1" OR "her1" OR "pEGFR" OR "phopho-EGFR") AND ("mouth" OR "oral" OR "oropharyn*") AND ("squamous cell carcinoma") AND ("transformation" OR "progression" OR "risk")) | 938 | Nov,  2024 |
| Total | 3869 | | |

**2. Supplementary Appendix S2. List of excluded studies with reasons (n = 27)**

**Cross-sectional (n = 19)**

1. Bagan J V., Mata-Roig M, Cortio-Gimeno J, Murillo-Cortes J, Hens-Aumente E, Poveda-Roda R, et al. Epidermal growth factor receptor copy number in potentially malignant oral disorders and oral squamous cell carcinoma: A short communication and preliminary study [Internet]. Vol. 41, Journal of Oral Pathology and Medicine. Denmark; 2012. p. 662–6. Available from: https://pubmed.ncbi.nlm.nih.gov/22417006/

2. Bánkfalvi A, Krassort M, Végh A, Felszeghy E, Piffkó J. Deranged expression of the E-cadherin/beta-catenin complex and the epidermal growth factor receptor in the clinical evolution and progression of oral squamous cell carcinomas. [Internet]. Vol. 31, Journal of oral pathology & medicine : official publication of the International Association of Oral Pathologists and the American Academy of Oral Pathology. Denmark; 2002. p. 450–7. Available from: https://pubmed.ncbi.nlm.nih.gov/12220351/

3. Chandran G, Balaram P, Kannan S, Pillai M, Nalinakumari K, Nair M. Immunohistochemical localization of epidermal growth-factor and its receptor in normal, premalignant and malignant oral-mucosa. Int J Oncol [Internet]. 1994 Feb [cited 2024 Nov 24];4(2):503–8. Available from: http://www.ncbi.nlm.nih.gov/pubmed/21566953

4. Cortés-Ramírez D, Rodríguez-Tojo M, Coca-Meneses J, Marichalar-Mendia X, Aguirre-Urizar J. Epidermal growth factor receptor expression in different subtypes of oral lichenoid disease. [Internet]. Vol. 19, Medicina oral, patologia oral y cirugia bucal. Spain; 2014. p. e451-8. Available from: https://pubmed.ncbi.nlm.nih.gov/24880441/

5. Ebrahimi M, Boldrup L, Wahlin YYB, Coates PJ, Nylander K, PJ C, et al. Decreased expression of the p63 related proteins beta-catenin, E-cadherin and EGFR in oral lichen planus. Oral Oncol [Internet]. 2008 Jul [cited 2024 Nov 24];44(7):634–8. Available from: https://pubmed.ncbi.nlm.nih.gov/17936670/

6. Huang HJH, Ping FFY, Hu JJA, Zhao SFS. [Expression of Notch1 and epidermal growth factor receptor in human tongue squamous cell carcinoma and precancerous lesion]. Hua Xi Kou Qiang Yi Xue Za Zhi [Internet]. 2009 Dec 1 [cited 2024 Nov 24];27(6):665-668,672. Available from: https://pubmed.ncbi.nlm.nih.gov/20077907/

7. Kannan S, GJ C, Balaram P, Chidambaram S, MK N. Potential biological markers for the staging of tumor progression in oral mucosa: a multivariate analysis. Int J Biol Markers [Internet]. 1996;11(2):67–76. Available from: https://pubmed.ncbi.nlm.nih.gov/8776606/

8. Kobayashi H, Kumagai K, Gotoh A, Eguchi T, Yamada H, Hamada Y, et al. Upregulation of epidermal growth factor receptor 4 in oral leukoplakia. [Internet]. Vol. 5, International journal of oral science. India; 2013. p. 14–20. Available from: https://pubmed.ncbi.nlm.nih.gov/23492901/

9. Kouhsoltani M, Aghbali A, Shokoohi B, Ahmadzadeh R. Molecular Targeting of Her-2/neu Protein Is Not Recommended as an Adjuvant Therapy in Oral Squamous Cell Carcinoma and Oral Lichen Planus. Adv Pharm Bull [Internet]. 2015;5:649–52. Available from: https://pubmed.ncbi.nlm.nih.gov/26793611/

10. Meka NJ, Ugrappa S, Velpula N, Kumar S, Maloth KN, Kodangal S, et al. Quantitative immunoexpression of egfr in oral potentially malignant disorders: Oral leukoplakia and oral submucous fibrosis. J Dent Res Dent Clin Dent Prospects [Internet]. 2015;9(3):166–74. Available from: https://pubmed.ncbi.nlm.nih.gov/26697149/

11. Moorthy A, DC V, Shyamsundar V, Madhavan Y, Ravindran S, Kuppuloganathan M, et al. Identification of EGFR as a Biomarker in Saliva and Buccal Cells from Oral Submucous Fibrosis Patients-A Baseline Study. Diagnostics (Basel, Switzerland) [Internet]. 2022;12(8). Available from: https://pubmed.ncbi.nlm.nih.gov/36010285/

12. Nagatsuka H, Ishiwari Y, Tsujigiwa H, Nakano K, Nagai N. Quantitation of epidermal growth factor receptor gene amplification by competitive polymerase chain reaction in pre-malignant and malignant oral epithelial lesions. Oral Oncol [Internet]. 2001;37(7):599–604. Available from: https://pubmed.ncbi.nlm.nih.gov/11564582/

13. Prado SMDS, Cedrún JLLJ, Rey RLR, Villaamil VMV, García AAÁ, Ayerbes MVM, et al. Evaluation of COX-2, EGFR, and p53 as biomarkers of non-dysplastic oral leukoplakias. [Internet]. Experimental and molecular pathology Netherlands; Oct, 2010 p. 197–203. Available from: https://pubmed.ncbi.nlm.nih.gov/20599939/

14. Rajeswari M, Saraswathi T. Expression of epithelial growth factor receptor in oral epithelial dysplastic lesions. J Oral Maxillofac Pathol [Internet]. 2012;16(2):183–8. Available from: https://pubmed.ncbi.nlm.nih.gov/22923888/

15. Shirasuna K, Hayashido Y, Sugiyama M, Yoshioka H, Matsuya T. Immunohistochemical localization of epidermal growth factor (EGF) and EGF receptor in human oral mucosa and its malignancy. Virchows Arch A Pathol Anat Histopathol [Internet]. 1991 Jul [cited 2024 Nov 24];418(4):349–53. Available from: https://pubmed.ncbi.nlm.nih.gov/2024456/

16. Singhal M, Tandon A, Juneja S, DC S, Kumar S, Jain A. Molecular Analysis as a Guide to Determining the Extent and Pathophysiology of Perilesional Tissues in Oral Epithelial Dysplasias. J Maxillofac Oral Surg [Internet]. 2020;19(3):447–55. Available from: https://pubmed.ncbi.nlm.nih.gov/32801543/

17. Srinivasan M, Jewell S. Quantitative estimation of PCNA, c-myc, EGFR and TGF-alpha in oral submucous fibrosis--an immunohistochemical study. Oral Oncol [Internet]. 2001;37(5):461–7. Available from: https://pubmed.ncbi.nlm.nih.gov/11377235/

18. Srinivasan M, Jewell SD. Evaluation of TGF-α and EGFR expression in oral leukoplakia and oral submucous fibrosis by quantitative immunohistochemistry. Oncology. 2001;61(4):284–92.

19. Zhao M, XL F, Lv H. The expression of EGFR in oral lichen planus, squamous cell papilloma and squamous cell carcinoma. Shanghai Kou Qiang Yi Xue [Internet]. 2012;21(6):673–6. Available from: https://pubmed.ncbi.nlm.nih.gov/23364554/

**Lack of essential data (n = 7)**

1. Beenken SW, Sellers MT, Huang P, Peters G, Krontiras H, Dixon P, et al. Transforming growth factor α (TGF-α) expression in dysplastic oral leukoplakia: Modulation by 13-cis retinoic acid. Head Neck. 1999 Sep;21(6):566–73.

2. Fu J, Chen W, Sun Z. [Gene expression of epidermal growth factor and epidermal growth factor receptor in oral lichen planus]. Zhonghua Kou Qiang Yi Xue Za Zhi. 2005;40(6):455–8.

3. Martín-Ezquerra G, Salgado R, Toll A, Gilaberte M, Baró T, Alameda Quitllet F, et al. Multiple genetic copy number alterations in oral squamous cell carcinoma: study of MYC, TP53, CCDN1, EGFR and ERBB2 status in primary and metastatic tumours. Br J Dermatol [Internet]. 2010 Nov [cited 2024 Nov 24];163(5):1028–35. Available from: https://pubmed.ncbi.nlm.nih.gov/20662834/

4. Mentrikoski MJ, Stelow EB, Culp S, Frierson HF, Cathro HP. Histologic and immunohistochemical assessment of penile carcinomas in a North American population. Am J Surg Pathol. 2014;38(10):1340–8.

5. Pospischil I, Hoetzenecker W. Drug eruptions with novel targeted therapies – immune checkpoint and EGFR inhibitors. JDDG - J Ger Soc Dermatology. 2021 Nov 1;19(11):1621–43.

6. Preti BTB, Pencz A, Cowger JJM, Vincent MD, Breadner D. Skin Deep: A Fascinating Case Report of Immunotherapy-Triggered, Treatment-Refractory Autoimmune Lichen Planus and Keratoacanthoma. Case Rep Oncol [Internet]. 2021 Aug 16 [cited 2024 Nov 24];14(2):1189–93. Available from: http://www.ncbi.nlm.nih.gov/pubmed/34703435

7. Singla S, Singla G, Zaheer S, Rawat DS, Mandal AK, DS R, et al. Expression of p53, epidermal growth factor receptor, c-erbB2 in oral leukoplakias and oral squamous cell carcinomas. J Cancer Res Ther [Internet]. 2018 Jan 1 [cited 2024 Nov 24];14(2):388–93. Available from: https://pubmed.ncbi.nlm.nih.gov/29516925/

**No oral cavity (n = 1)**

1. Krecicki T, Jeleń M, Zalesska-Krecicka M, Rak J, Szkudlarek T, Jeleń-Krzeszewska J. Epidermal growth factor receptor (EGFR), proliferating cell nuclear antigen (PCNA) and Ki-67 antigen in laryngeal epithelial lesions. Oral Oncol [Internet]. 1999;35(2):180–6. Available from: https://pubmed.ncbi.nlm.nih.gov/10435153/

**3. Supplementary Appendix S3. Characteristics of the included studies (n=9)**

| **Study**  **(year)** | **Country** | **Publication**  **language** | **Study design (recruitment period)** | **Follow up, m,**  **mean±SD**  **(range)** | **Patients with precancerous status and progression to cancer** | | | | | | | | **Analysis of EGFR protein expression** | | | | |
| --- | --- | --- | --- | --- | --- | --- | --- | --- | --- | --- | --- | --- | --- | --- | --- | --- | --- |
|  |  |  |  |  | **Sample**  **Size, n** | **Sex, M(%)/F, n;**  **age, y (mean±SD)** | **Tobacco,**  **n (%)** | **Alcohol,**  **n (%)** | **Affected**  **oral**  **subsites** | **OPMDs, n (diagnostic criteria)** | **Epithelial dysplasia, n (grading system)** | **Cancer development, n** | **Methods** | **EGFR antibody (dilution, incubation time, temperature)** | **IHC patern** | **IHC cutoff point (%)** | **EGFR(+),**  **n(%)** |
| Jäwert et al (2022) | Sweden | English | NR | Median: 102  NR | 23  Missing 5 | M=17 (60.7)  F=11 (39.3)  Median 68.5  Range=39-93 | NR | NR | Tongue: 12  Bm: 9  Gingiva:6  FOM: 1 | Leukoplakia  23 | No ED: 6  Mild: 8  Moderate: 10  Severe: 4  (NR) | 9 | FISH | NR | NR | NR | 10  (43.48) |
| Monteiro et al (2022) | Portugal | English | Retrospective  cohort  (1995-2006) | 32.4±29  (2-120) | 45  Missing 19 | M=46 (71.9)  F=18 (28.1)  58.1 ±16.8  NR | 17  (40.5) | 15  (56.2) | Tongue: 27  Bm: 13  Gingiva:11  FOM: 2  Palate: 1  Others: 10 | Leukoplakia  52 | Low grade: 41  High grade: 11  (binary histological grade clasification) | 5 | IHC | Clone EGFR.25  (1:100, NR  NR) | Membranous | Labelling index =  extent x  Intensity | 43  (95.5) |
| Tarle et al. (2022) | Croatia | English | Retrospective cohort  (2010-2014) | Median: 63.6  (25.2-134.4) | 50 | M=23(47.62)  F=27(52.38)  64.22±14.35  NR | 36  (76.19) | 21  (42) | Tongue:21  Bm:6  Gingiva: 3  Palate:6  Others: 14 | Leukoplakia  31  Erythroplakia  19 | No ED:15  Mild:13  Moderate:5  Severe:17  (WHO NR) | 21 | IHC | Clone EGFR-1  (1:25  90’,NR) | Nuclear | 18 | 24  (48) |
| Ries et al. (2017) | Germany | English | Retrospective  cohort  (1997-2011) | NR  (NR-60) | 98 | M=59 (60.2)  F=39 (39.8)  55.8  NR | NR | NR | NR | Leukoplakia  (NR) | No ED:37  Mild:33  Moderate:17  Severe:11  (WHO 2005) | 53 | IHC | antibody M3563 (1:300)  NR | Membranous  Cytoplasmic | 44.96 | 38  (38.77) |
| Nankivel et al. (2013) | United Kingdom | English | Retrospective  cohort  (1996-2008) | NR  (NR-120) | 148 | M=76 (51.36)  F=72 (48.64)  61±13.6  (19-90) | 78  (52.70) | 81  (54.73) | Tongue: 69  Bm: 38  FOM: 20  Palate 18  Others: 3 | Leukoplakia  107  Erythroplakia  15 Others  26 | No ED: 0  Mild: 69  Moderate: 50  Severe/cis: 29  (WHO NR) | 39 | IHC | NR | Membranous | NR | NR |
| Poh et al. (2011) | Canada | English | NR | NR | 35 | NR | NR | NR | NR | NR | NR | 22 | FISH | NR | NR | NR | 20  (57.14) |
| Bencheckroun et al. (2010) | United States | English | Prospective  (1992-2001) | Median:90  NR | 145 | M=77 (53.10)  F=68 (48.90)  NR  NR | 109  (75.17) | 99  (68.27) | NR | NR | No ED: 97  Mild: 37  Moderate-Severe:11  (NR) | 35 | IHC | Clone 31G7  (1:100,  90’, RT) | Membranous_ | >10% | 103 |
| Bencheckroun et al. (2010) | United States | English | Prospective  (1992-2001) | Median:90  NR | 49 | M=26 (53.06)  F=23 (46.94)  NR  NR | 41  (83.67) | 37  (75.51) | NR | NR | No ED:33  Mild: 10  Moderate-Severe:6  (NR) | 24 | FISH | NR | NR | NR | 20 |
| Shah et al. (2007) | India | English | Retrospective cohort  (2000-2003) | NR | 60 | M=54 (90)  F=6 (10)  NR  (18-75) | 53  (88.33) | NR | Bm:60 | Leukoplakias  45  Submoucous fibrosis  15 | No ED:35  Mild:16  Moderate:5  Severe:4  (NR) | NR | IHC | Clone 111.6  (1:50,  overnight, 4ºC) | Membranous  Cytoplasmic | 10 | 15  (25) |
| Abbreviations: m, months; SD, standard deviation; M, male; F, female; n, sample size; OPMD, oral potentially malignant disorders; EGFR, epidermal growth factor receptor; IHC, immunohistochemistry; NR, not reported; Bm, buccal mucosa; FOM, floor of mouth; ED, oral epithelial dysplasia; WHO, World Health Organization;  IHC, immunohistochemistry; FISH, fluorescence in situ hybridization; RT, room temperature; ºC, Celsius. | | | | | | | | | | | | | | | | | |

**4. Supplementary Appendix S4. Meta-analysis on the association between EGFR upregulation and OPMDs malignant transformation risk**

**4.1 Subgroup analysis by alteration**

**Figure S1.** Forest plot graphically representing the stratified meta-analysis on the association between EGFR upregulation and OPMDs malignant transformation risk by type of alteration (i.e., EGFR protein overexpression vs *EGFR* gene amplification).


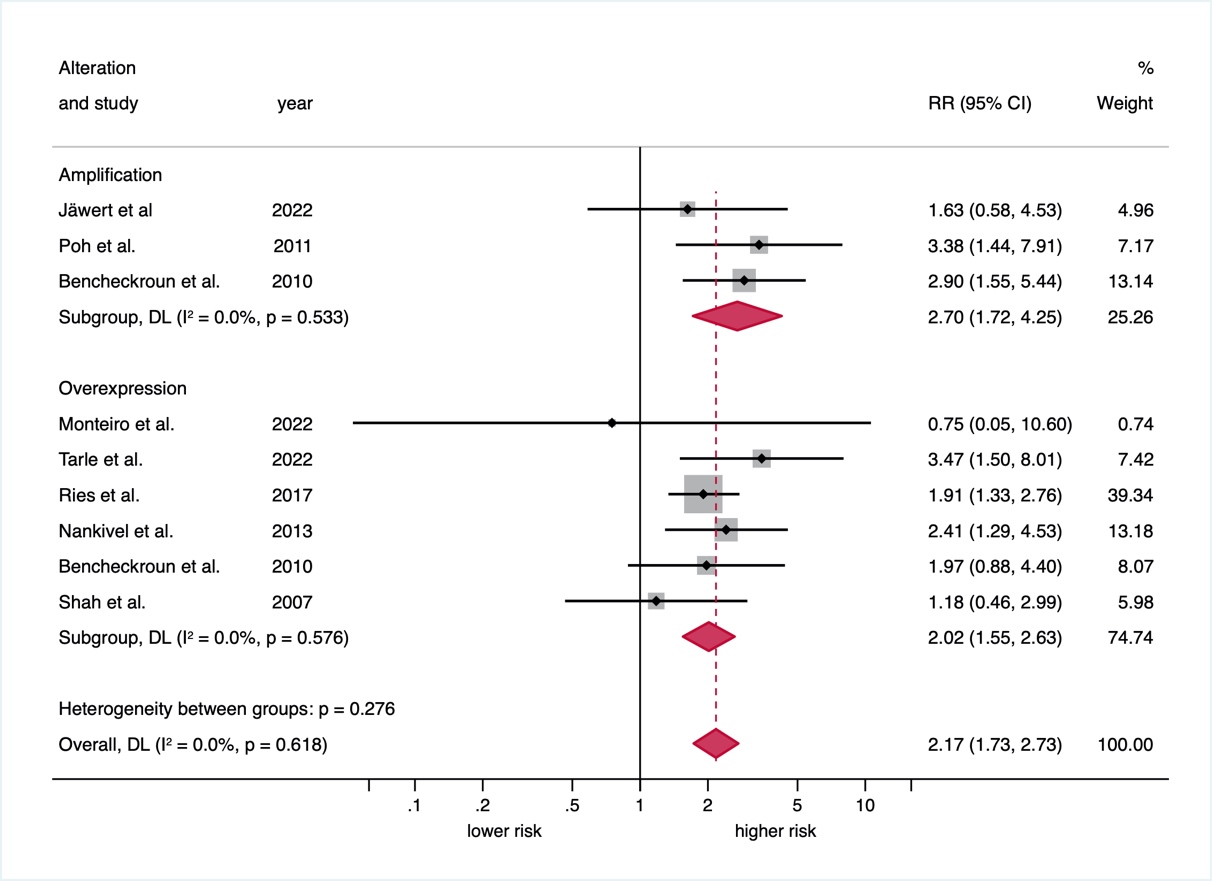


RR, relative risk; CI, confidence intervals, DerSimonian and Laird, DL. Random-effects model, inverse-variance weighting based on the DL method. A RR > 1 suggests that EGFR upregulation is associated with a higher malignant transformation risk. Diamonds indicate the pooled RR with their corresponding 95% CIs.

**4.2 Subgroup analysis by geographical region**

**Figure S2.** Forest plot graphically representing the stratified meta-analysis on the association between EGFR upregulation and OPMDs malignant transformation risk by geographical region.

**
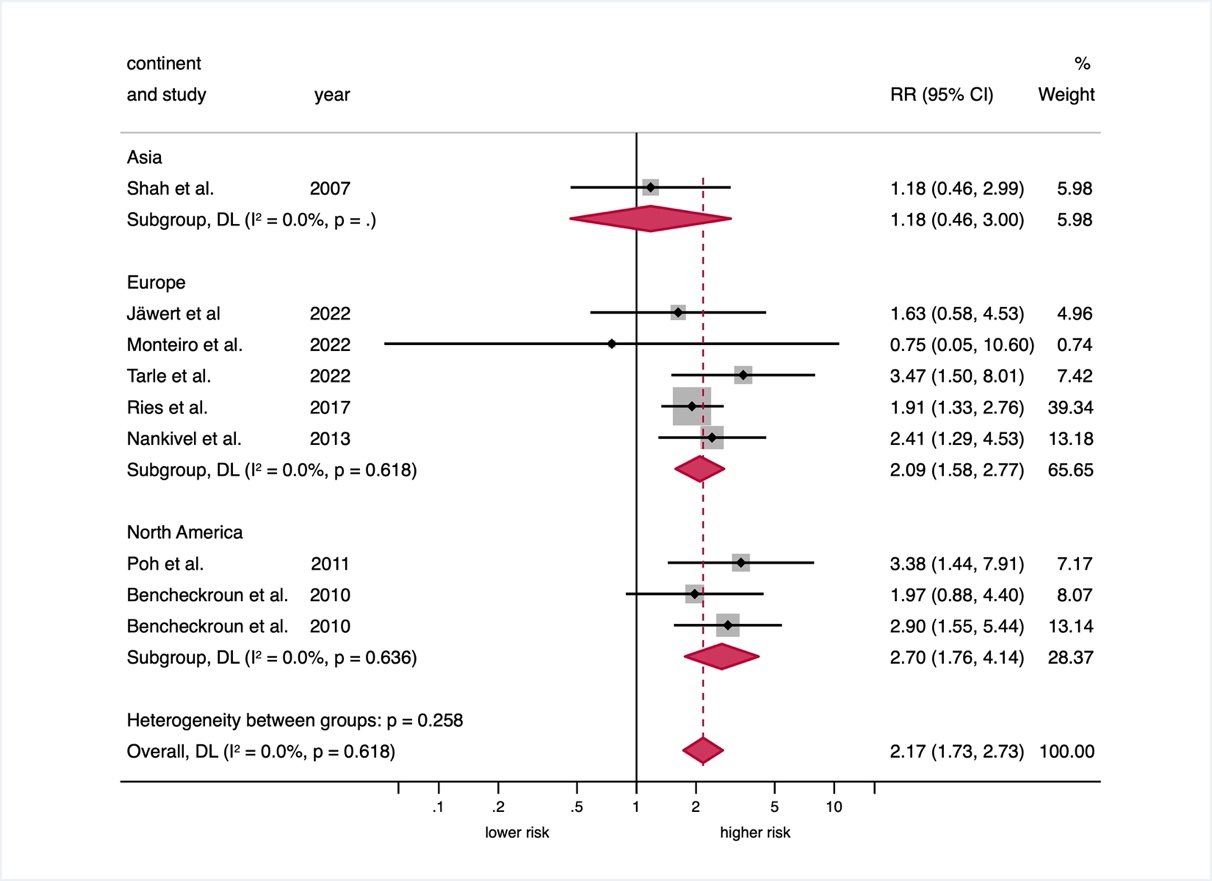
**

RR, relative risk; CI, confidence intervals, DerSimonian and Laird, DL. Random-effects model, inverse-variance weighting based on the DL method. A RR > 1 suggests that EGFR upregulation is associated with a higher malignant transformation risk. Diamonds indicate the pooled RR with their corresponding 95% CIs.

**4.3 Subgroup analysis by type of OPMD**

**Figure S3.** Forest plot graphically representing the stratified meta-analysis on the association between EGFR upregulation and OPMDs malignant transformation risk by type of OPMD.

**
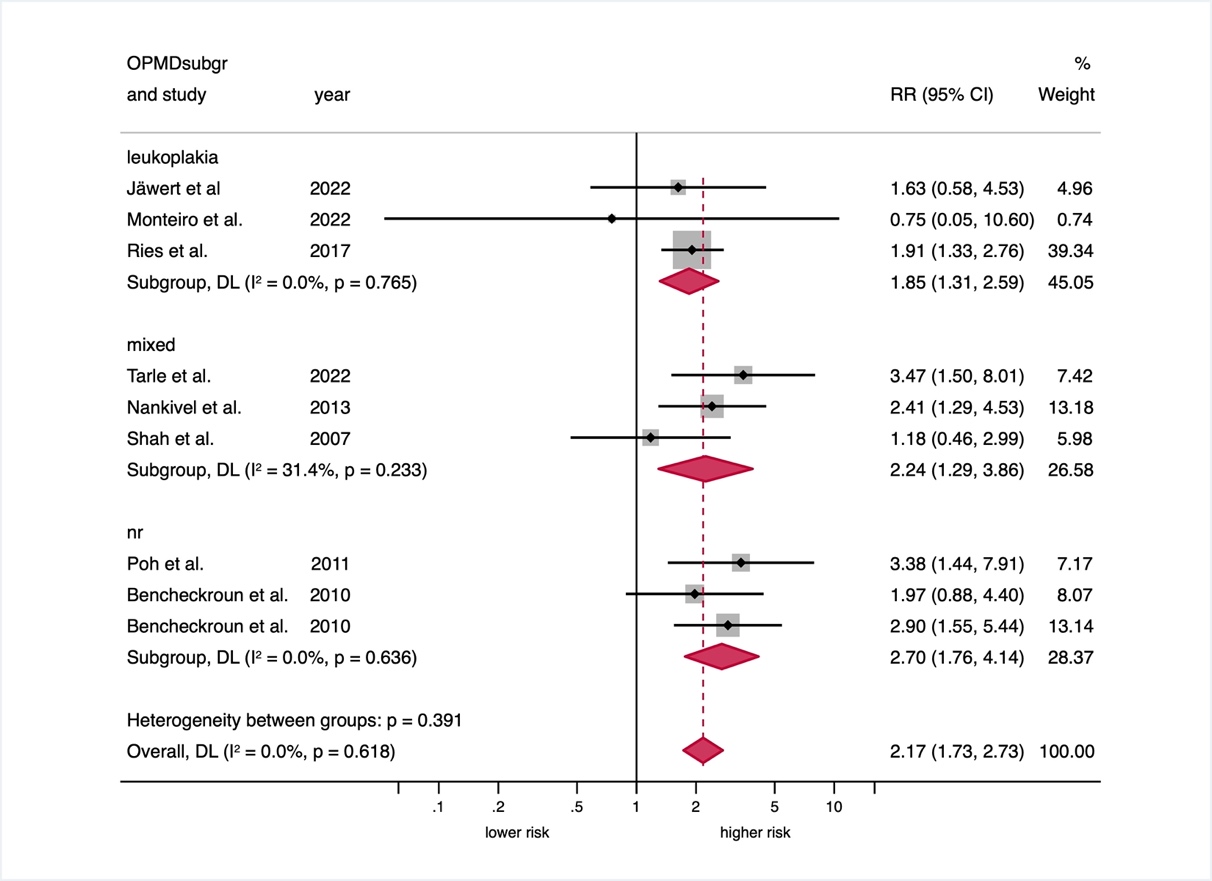
**

RR, relative risk; CI, confidence intervals, DerSimonian and Laird, DL; nr, not reported. Random-effects model, inverse-variance weighting based on the DL method. A RR > 1 suggests that EGFR upregulation is associated with a higher malignant transformation risk. Diamonds indicate the pooled RR with their corresponding 95% CIs.

**4.4 Subgroup analysis by immunohistochemical pattern**

**Figure S4.** Forest plot graphically representing the stratified meta-analysis on the association between EGFR upregulation and OPMDs malignant transformation risk by immunohistochemical pattern.

**
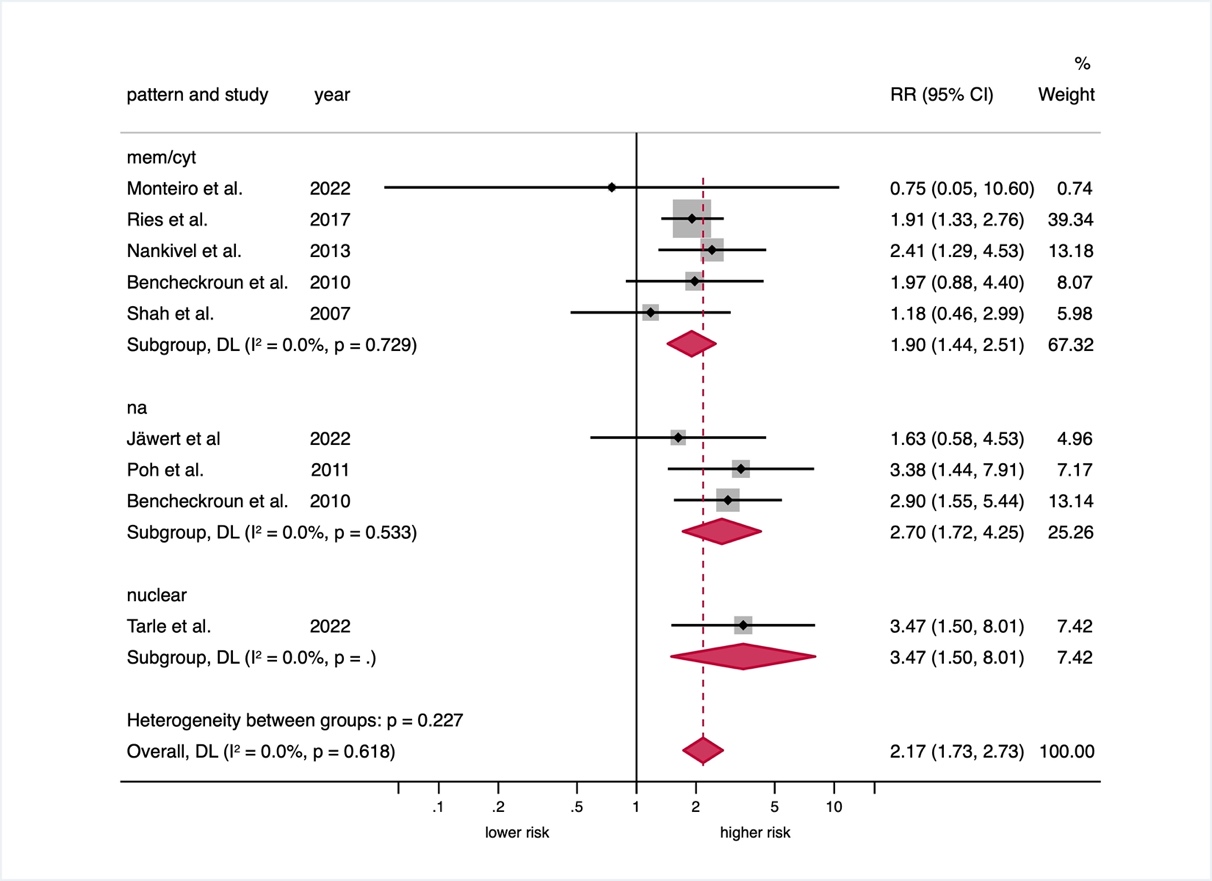
**

RR, relative risk; CI, confidence intervals, DerSimonian and Laird, DL. Random-effects model, inverse-variance weighting based on the DL method; na, not applicable. A RR > 1 suggests that EGFR upregulation is associated with a higher malignant transformation risk. Diamonds indicate the pooled RR with their corresponding 95% CIs.

**4.5 Subgroup analysis by anti-EGFR antibody**

**Figure S5.** Forest plot graphically representing the stratified meta-analysis on the association between EGFR upregulation and OPMDs malignant transformation risk by anti-EGFR antibody.


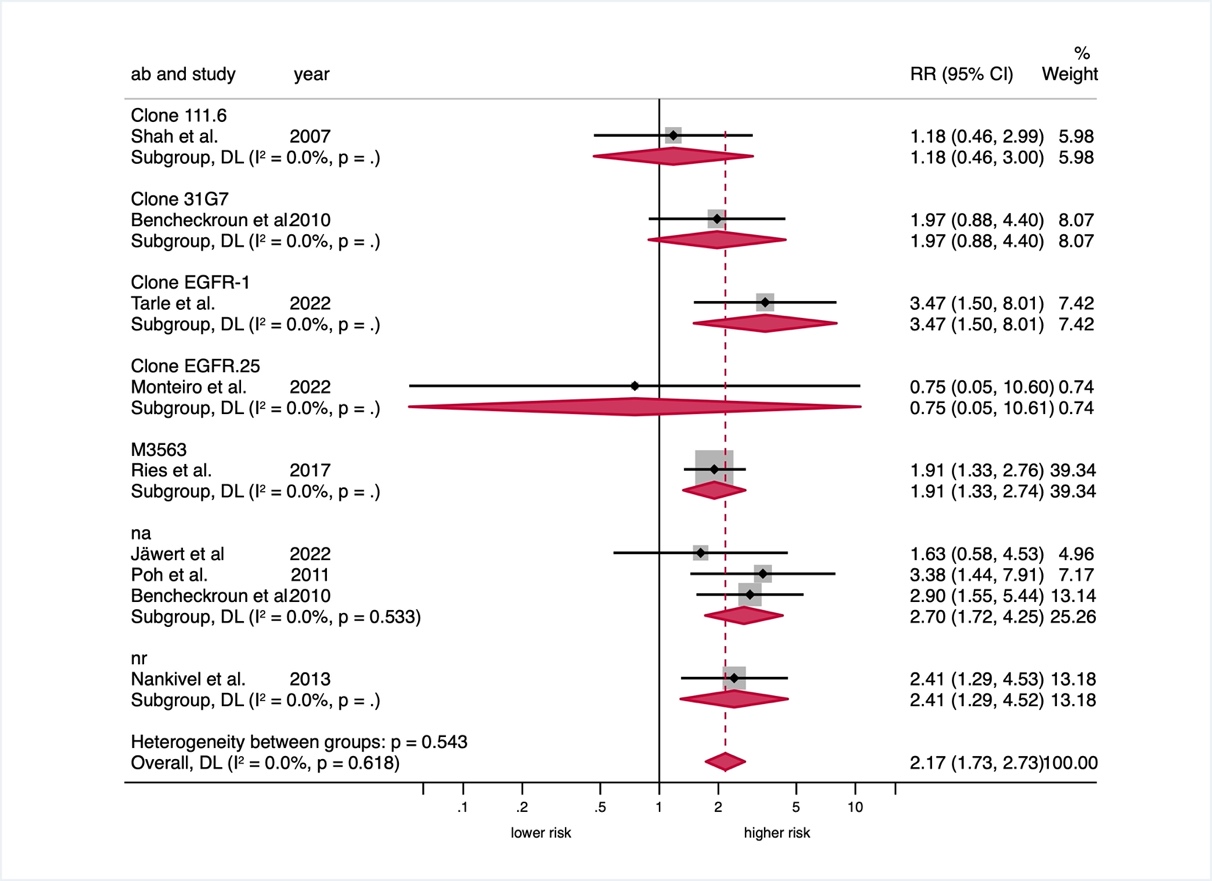


RR, relative risk; CI, confidence intervals, DerSimonian and Laird, DL; nr, not reported; na, not applicable. Random-effects model, inverse-variance weighting based on the DL method. A RR > 1 suggests that EGFR upregulation is associated with a higher malignant transformation risk. Diamonds indicate the pooled RR with their corresponding 95% CIs.

**4.6 Subgroup analysis by anti-EGFR antibody dilution**

**Figure S6.** Forest plot graphically representing the stratified meta-analysis on the association between EGFR upregulation and OPMDs malignant transformation risk by anti-EGFR antibody dilution.


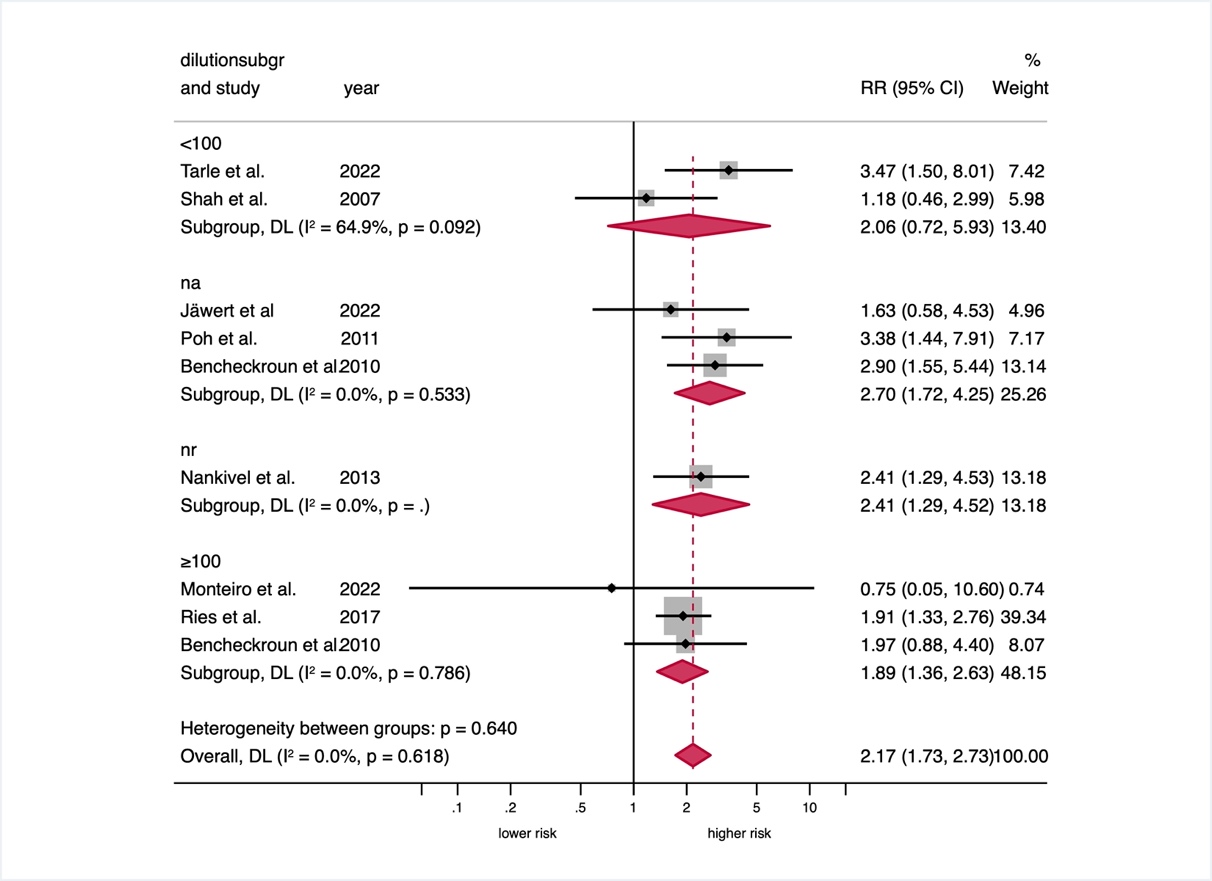


RR, relative risk; CI, confidence intervals, DerSimonian and Laird, DL; nr, not reported; na, not applicable. Random-effects model, inverse-variance weighting based on the DL method. A RR > 1 suggests that EGFR upregulation is associated with a higher malignant transformation risk. Diamonds indicate the pooled RR with their corresponding 95% CIs.

**4.7 Subgroup analysis by anti-EGFR antibody incubation time**

**Figure S7.** Forest plot graphically representing the stratified meta-analysis on the association between EGFR upregulation and OPMDs malignant transformation risk by anti-EGFR antibody incubation time.


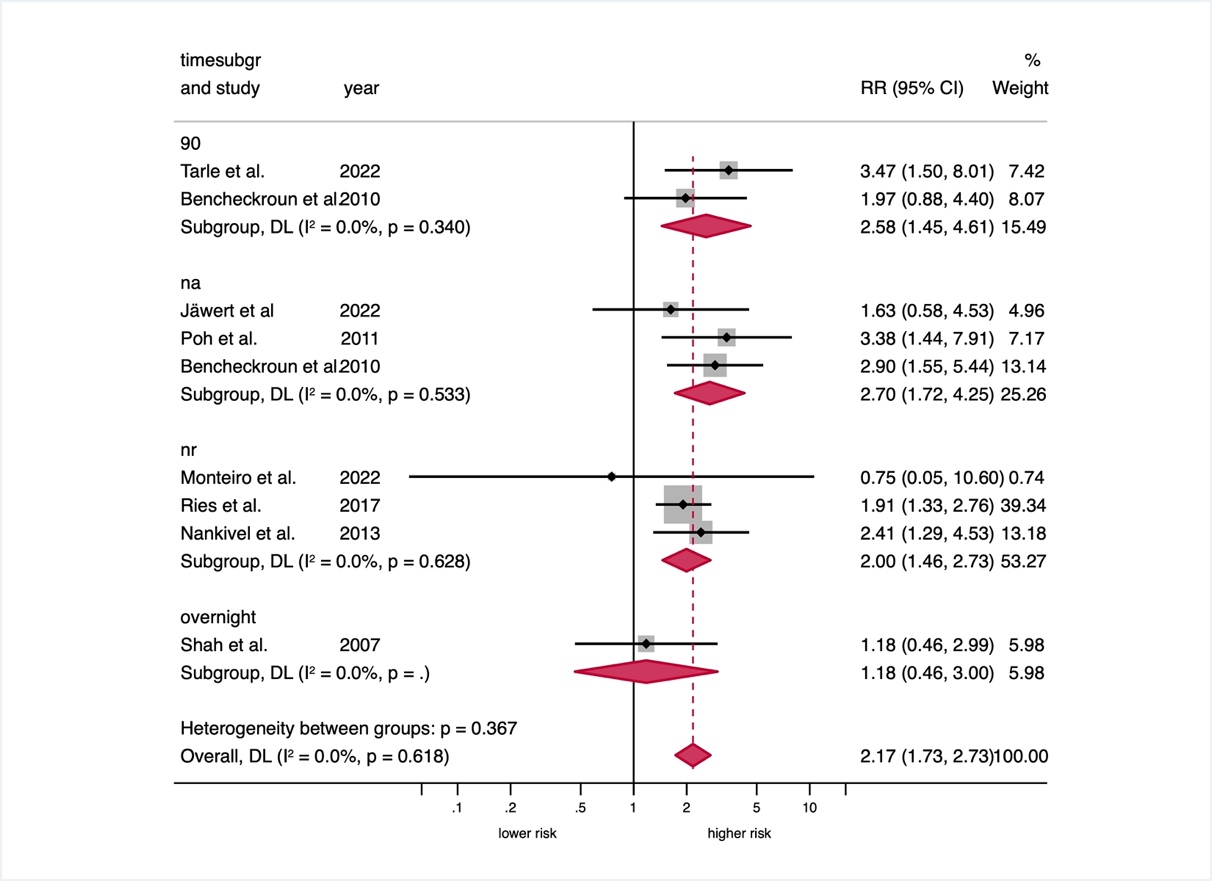


RR, relative risk; CI, confidence intervals, DerSimonian and Laird, DL; nr, not reported; na, not applicable. Random-effects model, inverse-variance weighting based on the DL method. A RR > 1 suggests that EGFR upregulation is associated with a higher malignant transformation risk. Diamonds indicate the pooled RR with their corresponding 95% CIs.

**4.8 Subgroup analysis by anti-EGFR antibody incubation temperature**

**Figure S8.** Forest plot graphically representing the stratified meta-analysis on the association between EGFR upregulation and OPMDs malignant transformation risk by anti-EGFR antibody incubation temperature.


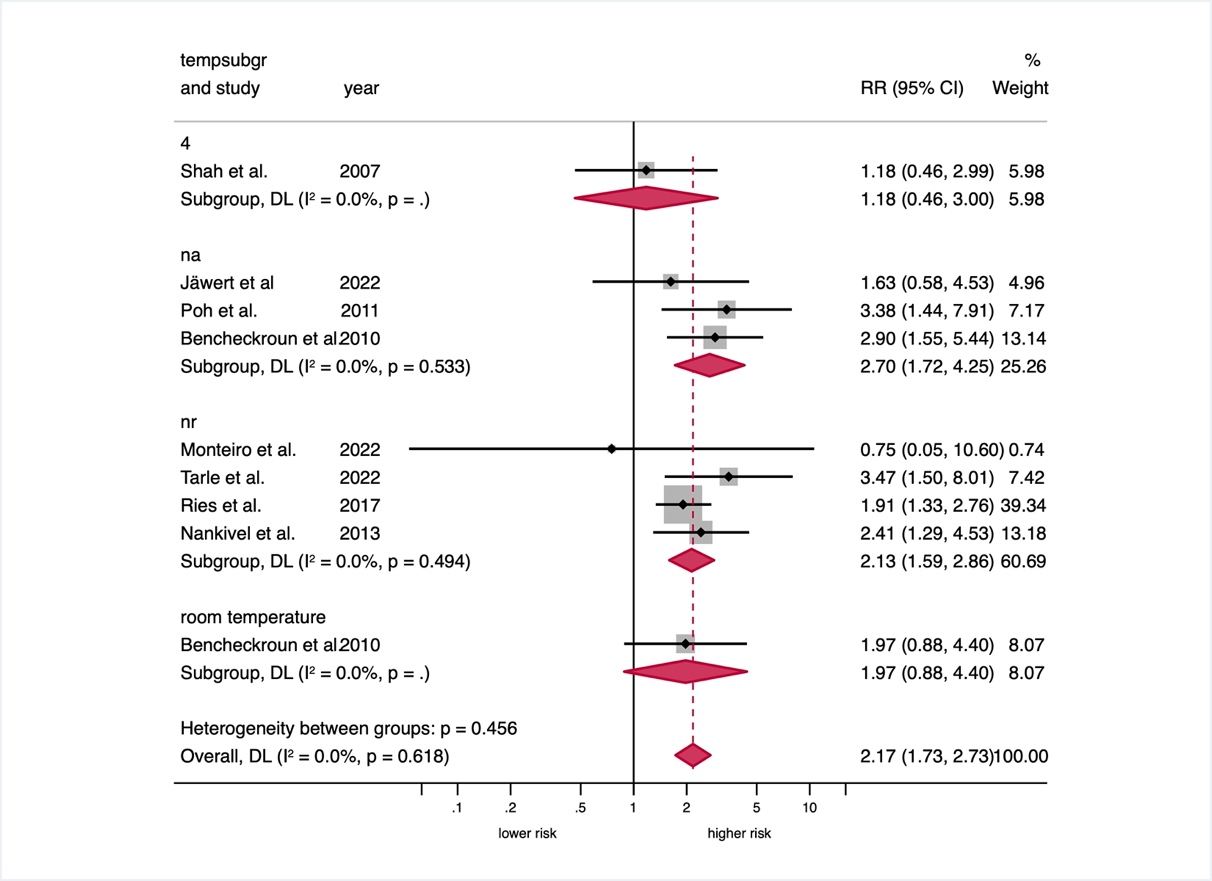


RR, relative risk; CI, confidence intervals, DerSimonian and Laird, DL; nr, not reported; na, not applicable. Random-effects model, inverse-variance weighting based on the DL method. A RR > 1 suggests that EGFR upregulation is associated with a higher malignant transformation risk. Diamonds indicate the pooled RR with their corresponding 95% CIs.

**4.9 Subgroup analysis by cutoff point for EGFR overexpression**

**Figure S9.** Forest plot graphically representing the stratified meta-analysis on the association between EGFR overexpression and OPMDs malignant transformation risk by cutoff point for EGFR overexpression.

**
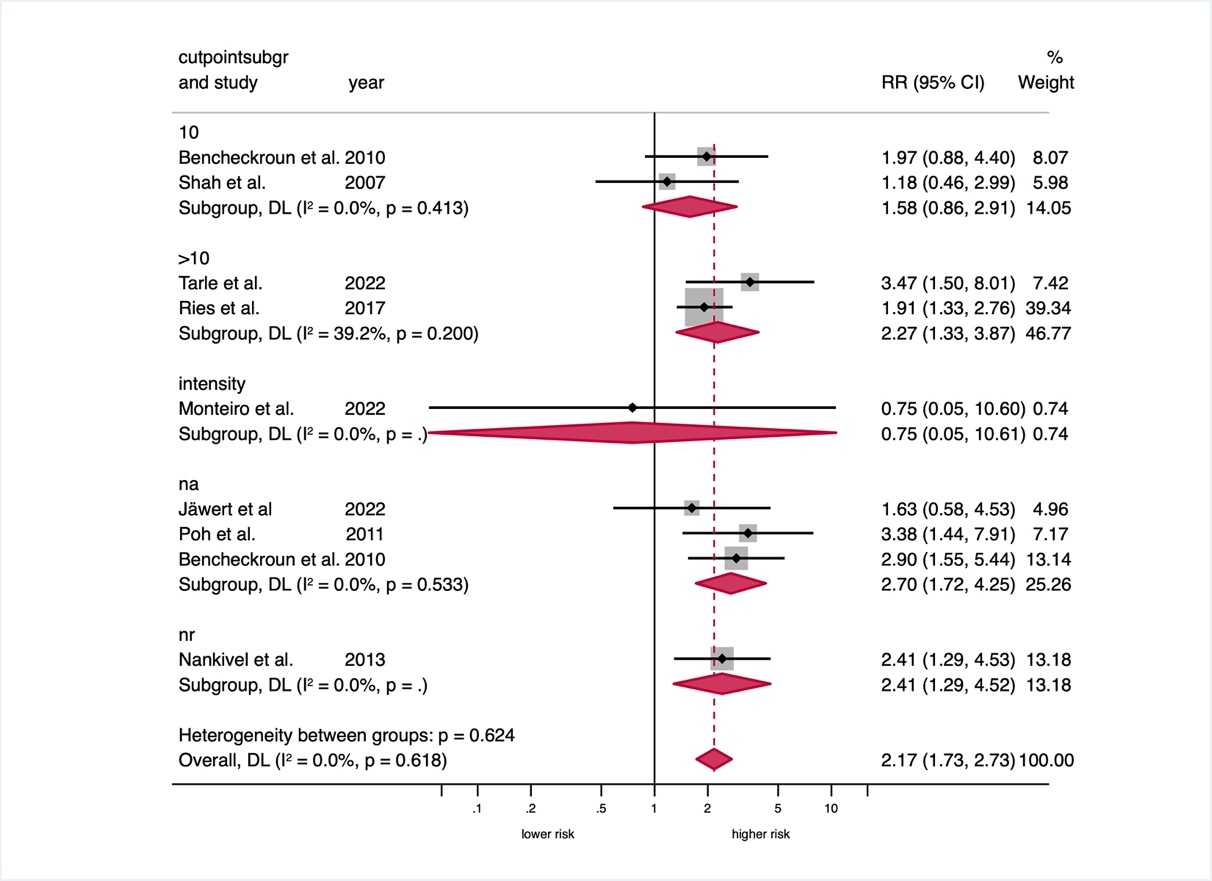
**

RR, relative risk; CI, confidence intervals, DerSimonian and Laird, DL; nr, not reported; na, not applicable. Random-effects model, inverse-variance weighting based on the DL method. A RR > 1 suggests that EGFR overexpression is associated with a higher malignant transformation risk. Diamonds indicate the pooled RR with their corresponding 95% CIs.

**4.10 Subgroup analysis by overall risk of bias in primary-level studies**

**Figure S10.** Forest plot graphically representing the stratified meta-analysis on the association between EGFR upregulation and OPMDs malignant transformation risk by overall risk of bias in primary-level studies.

**
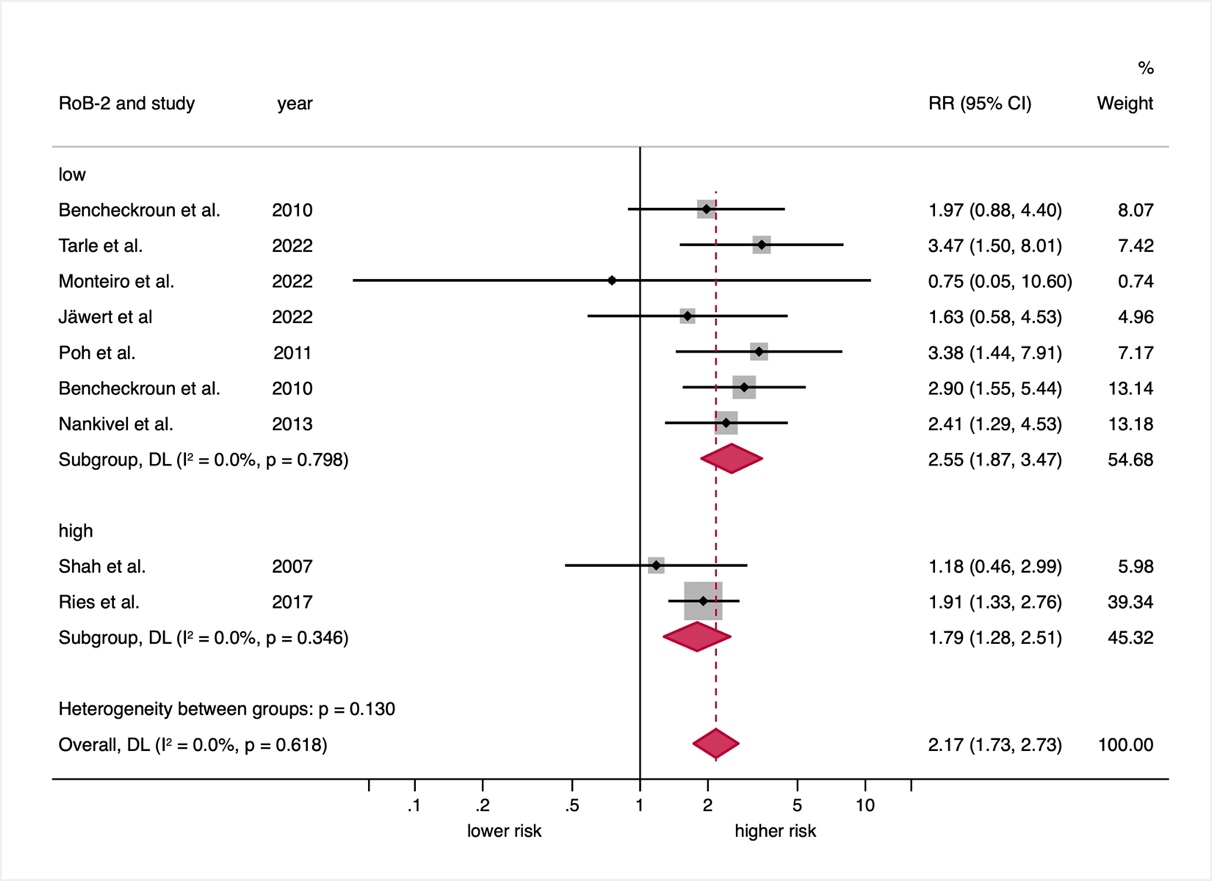
**

RR, relative risk; CI, confidence intervals, DerSimonian and Laird, DL. Random-effects model, inverse-variance weighting based on the DL method. A RR > 1 suggests that EGFR upregulation is associated with a higher malignant transformation risk. Diamonds indicate the pooled RR with their corresponding 95% CIs.

**5. Supplementary Appendix S5. Analysis of small‐study effects**

**Figure S11.** A funnel plot of estimated logRRs against their SE, graphically representing the analysis of small-study effects on the association between EGFR upregulation and OPMDs malignant transformation risk.

**
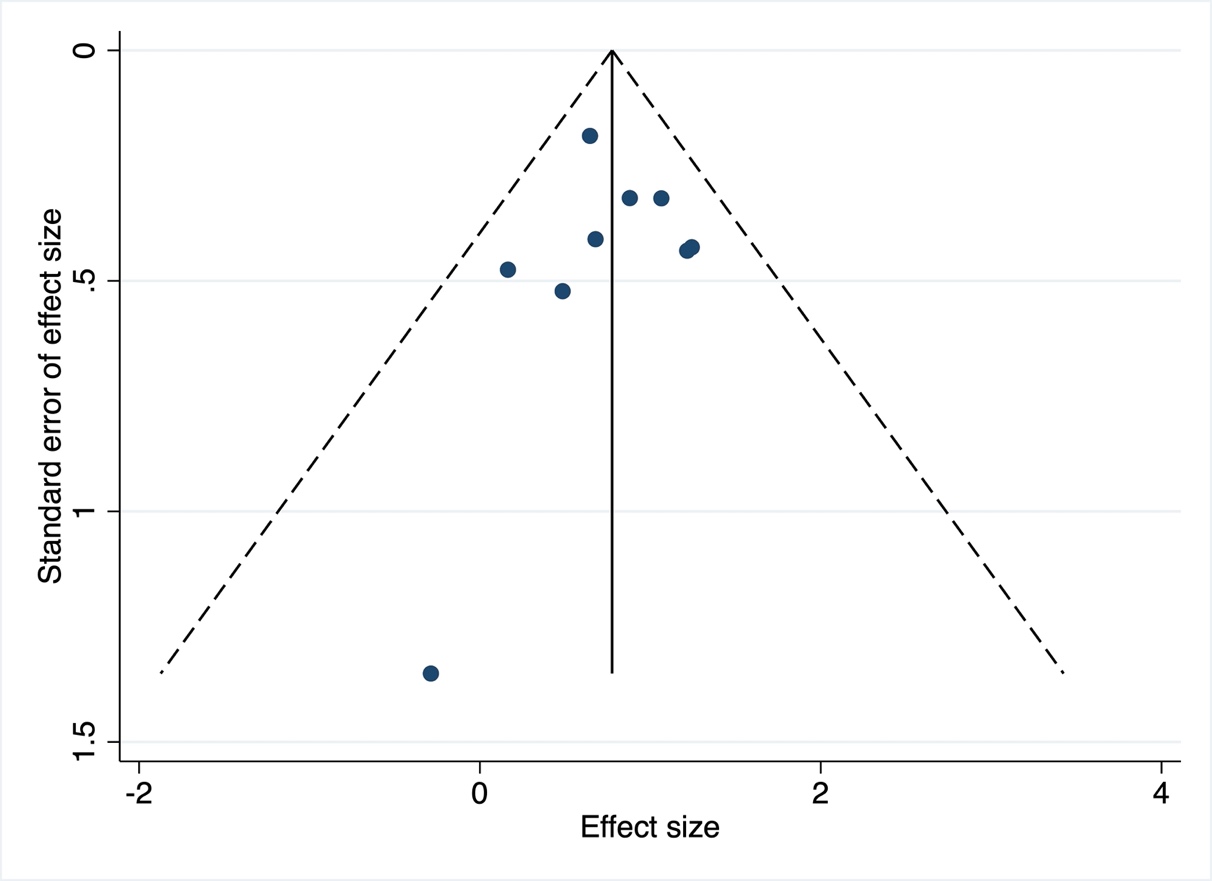
**

SE, standard error; RR, relative risk; log, natural logarithm (i.e., log base e). The black vertical line corresponds to the pooled effect size estimated in the meta-analysis. The two diagonal intermittent lines represent the pseudo-95% confidence interval. The blue circles represent the estimates from primary-level studies.

**6. Supplementary Appendix S6. Sensitivity analysis (leave-one-out method).**

Figure S12. Interval plot graphically representing the sensitivity analysis of the studies pooled in the meta-analysis on the association between EGFR upregulation and OPMDs malignant transformation risk.


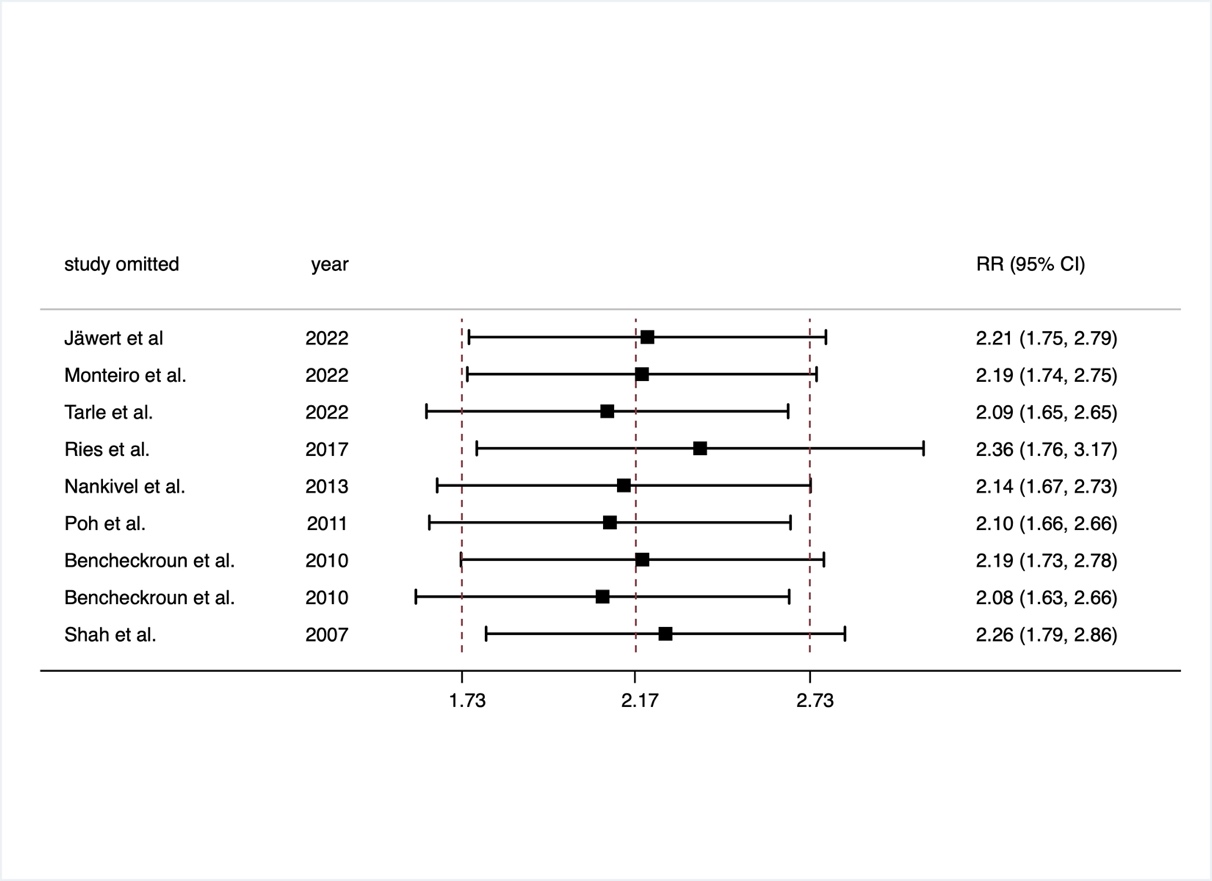


RR, relative risk; CI, confidence intervals. Sensitivity analysis (“leave-one-out” method) of the meta-analysis results, sequentially omitting one study at a time to investigate its influence on the overall result. In the interval plot, the usual diamond shape representing the pooled effect was replaced by vertical intermittent red lines, allowing a visual inspection analysis of influence.
